# Transmission-Blocking Antibodies against Mosquito C-Type Lectins for Dengue Prevention

**DOI:** 10.1371/journal.ppat.1003931

**Published:** 2014-02-13

**Authors:** Yang Liu, Fuchun Zhang, Jianying Liu, Xiaoping Xiao, Siyin Zhang, Chengfeng Qin, Ye Xiang, Penghua Wang, Gong Cheng

**Affiliations:** 1 Department of Basic Medical Sciences, School of Medicine, Tsinghua University, Beijing, People's Republic of China; 2 Guangzhou 8th People's Hospital, Guangzhou, People's Republic of China; 3 State Key Laboratory of Pathogen and Biosecurity, Beijing Institute of Microbiology and Epidemiology, Beijing, People's Republic of China; 4 Section of Infectious Diseases, Yale University School of Medicine, New Haven, Connecticut, United States of America; 5 Collaborative Innovation Center for Diagnosis and Treatment of Infectious Diseases, Hangzhou, People's Republic of China; Washington University School of Medicine, United States of America

## Abstract

C-type lectins are a family of proteins with carbohydrate-binding activity. Several C-type lectins in mammals or arthropods are employed as receptors or attachment factors to facilitate flavivirus invasion. We previously identified a C-type lectin in *Aedes aegypti*, designated as mosquito galactose specific C-type lectin-1 (*mosGCTL-1*), facilitating the attachment of West Nile virus (WNV) on the cell membrane. Here, we first identified that 9 *A. aegypti mosGCTL* genes were key susceptibility factors facilitating DENV-2 infection, of which *mosGCTL-3* exhibited the most significant effect. We found that *mosGCTL-3* was induced in mosquito tissues with DENV-2 infection, and that the protein interacted with DENV-2 surface envelop (E) protein and virions *in vitro* and *in vivo*. In addition, the other identified mosGCTLs interacted with the DENV-2 E protein, indicating that DENV may employ multiple mosGCTLs as ligands to promote the infection of vectors. The vectorial susceptibility factors that facilitate pathogen invasion may potentially be explored as a target to disrupt the acquisition of microbes from the vertebrate host. Indeed, membrane blood feeding of antisera against mosGCTLs dramatically reduced mosquito infective ratio. Hence, the immunization against mosGCTLs is a feasible approach for preventing dengue infection. Our study provides a future avenue for developing a transmission-blocking vaccine that interrupts the life cycle of dengue virus and reduces disease burden.

## Author Summary

Dengue virus (DENV), a mosquito-borne flavivirus, is currently the most significant arbovirus afflicting tropical and sub-tropical countries worldwide. No vaccine or therapeutics are available, and dengue has rapidly spread over the last decade. Therefore, additional strategies to combat dengue are urgently needed. In this study, we characterized multiple C-type lectins as susceptibility factors for dengue infection in *A. aegypti*. These mosGCTLs directly interacted with dengue virus *in vitro* and *in vivo*. The combination of antisera against multiple mosGCTLs efficiently reduced DENV-2 infection after a blood meal, suggesting that it is feasible to develop a mosGCTL-based transmission-blocking vaccine to interrupt the life cycle of dengue virus and control disease burden in nature. This study substantially extends our understanding of dengue replication in vectors and provides a research avenue by which the development of therapeutics for preventing the dissemination of mosquito-borne viral diseases can be pursued in the future.

## Introduction

Dengue virus (DENV) is a mosquito-borne flavivirus belonging to the *Flaviviridae* family. There are 4 serotypes of dengue virus (DENV-1 to DENV-4) that cause human diseases by transmission via the mosquito vector *Aedes spp.* [1], [2]. DENV infections in humans result in a broad spectrum of clinical symptoms, ranging from mild fever (dengue fever) to dengue hemorrhagic fever, the latter of which can progress to dengue shock syndrome and death [1]. Dengue-related diseases have been shown to be a major global medical problem. There are more than 100 dengue-endemic countries in the world. Further, approximately 2.5 billion people have a risk of dengue infection every year. The World Health Organization (WHO) estimates that there are around 50 million dengue infections per year, of which approximately 500,000 lead to severe clinical symptoms that require hospitalization and more than 20,000 cases lead to death, mostly in tropical countries [3], [4]. In 2010, 1.6 million cases of dengue were reported in the Americas alone, of which 49,000 cases involved severe hemorrhagic-related clinical symptoms (www.who.int/mediacentre/factsheets/fs117/en/). There are no dengue vaccines or therapeutics available, and therefore, novel preventive approaches are urgently needed to combat DENV infection.

*A. aegypti* that belongs to the *Aedes* genus is a dominant species for DENV transmission [5]. The genome of *A. aegypti* has been characterized, and this significantly increased our understanding of flaviviral pathogenesis and the transmission mechanisms of mosquito-borne microbes [6], [7], [8], [9]. As an anthropophilic vector in and around human dwellings, *A. aegypti* is simple to cultivate and is readily susceptible to dengue virus in the laboratory. The virus rapidly disseminates throughout the mosquito tissues after a blood meal or intrathoracic microinjection [9], [10], [11], [12]. Dengue virus is transmitted from the *Aedes spp.* to humans during vector engorgement [13], [14]. Therefore, approaches that interrupt the life cycle of dengue virus may efficiently reduce the number of infected mosquitoes and help to control future dengue dissemination.

C-type lectins are a family of proteins with carbohydrate-binding activity that have been shown to have vital roles in immune activation and viral pathogenesis [15]. Human mannose-binding lectins (MBL) bind to glycans on dengue surface envelope (E) protein, leading to the activation of complement immune cascades [16], [17]. In contrast, several mammalian C-type lectins are employed as receptors or attachment factors to facilitate dengue invasion. DC-SIGN (CD209) binds to the dengue virus via high-mannose glycans on the dengue E protein, and it is an essential attachment factor for the invasion of dendritic cells [18], [19], [20], [21]. The mannose receptor (MR), another C-type lectin, is expressed on macrophages and interacts with the dengue E protein to enhance viral attachment to phagocytes [22]. Besides facilitating viral attachment and entry, C-type lectins also play a role in regulating immune signaling during dengue infection. C-type lectin domain family 5, member A (CLEC5A) had been found to be associated with dengue virus [23]. The binding does not result in viral entry, but rather stimulates the release of pro-inflammatory cytokines, potentially contributing to the pathogenesis of dengue hemorrhagic fever [23]. The C-type lectins in mosquitoes also play crucial roles in flaviviral infection. We previously identified a C-type lectin in *A. aegypti*, designated as mosquito galactose specific C-type lectin-1 (mosGCTL-1), that serves as a soluble receptor for facilitating the attachment of West Nile virus (WNV) via an association with mosPTP-1 on the cell membrane. However, *mosGCTL-1* silencing did not influence DENV-2 infection of *A. aegypti* [9].

For arthropod-borne microbes, vector ligands that interact with pathogens are potential targets for interfering with the successful acquisition of the microbe from the vertebrate host. For instance, blocking the tick gut receptor for the Lyme disease agent limits the colonization of ticks by *Borrelia burgdorferi* [24]. Therefore, a better understanding of flaviviral infection in vectors may lead to the identification of novel targets for preventive strategies. Our previous studies showed that uptake of mosGCTL-1 antisera dramatically interrupts the infection with West Nile virus (WNV) during a blood meal [9], indicating that a humoral response in the host against the vector ligand reduced the vectorial capacity for infection and interrupted the arboviral life cycle. Given the close relationship between WNV and DENV, we presumed that some *mosGCTLs* may also facilitate DENV infection. Here, using *in vivo* RNA interference (RNAi) screening, we identified 9 of the 36 genes in the *mosGCTL* family that contribute to DENV-2 infection of *A. aegypti*. Among the identified *mosGCTL* genes, *mosGCTL-3* exhibited the most significant effect. Therefore, we used *mosGCTL-3* to explore the role of the *mosGCTL* family in DENV infection. Consistent with the role of mosGCTL-1 in WNV infection, mosGCTL-3 interacted with DENV-2 *in vivo* and *in vitro* to enhance the infection in *A. aegypti*. Importantly, membrane blood feeding of antisera against mosGCTLs dramatically reduced DENV-2 infection of mosquitoes. These findings will help in directing future development of a transmission-blocking vaccine that can interrupt the dengue life cycle and contribute to dengue prevention.

## Results

### The role of the *mosGCTLs* family in the infection of *A. aegypti* with DENV

**Figure 1:**
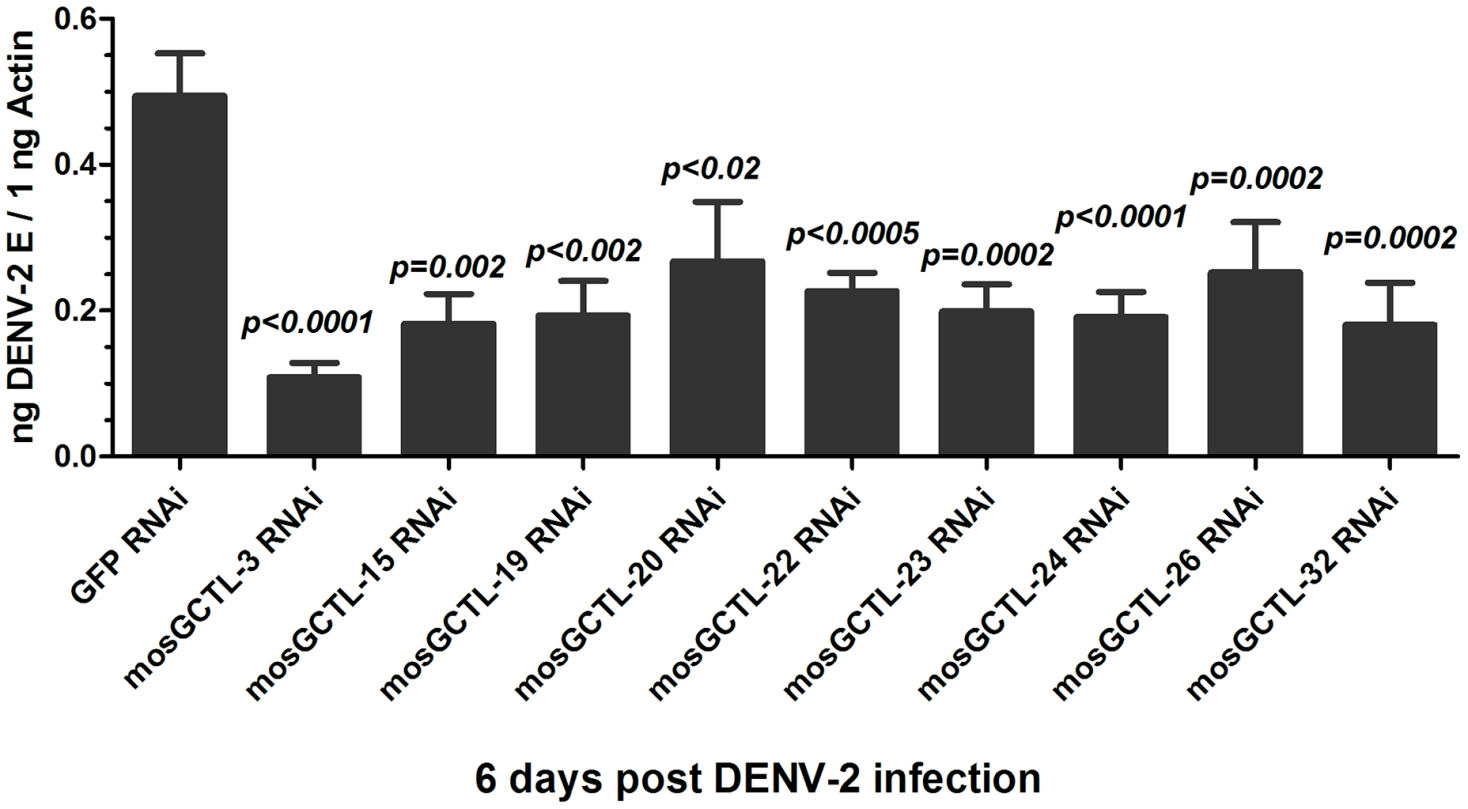
The role of *mosGCTL* genes in DENV-2 infection of *A. aegypti*. The phenotype, of which the 9 *mosGCTL* genes exhibited susceptible effect in DENV-2 (New Guinea C strain) infection (*p<0.05*, Table S1), was reproduced. There were no less than 15 mosquitoes in each group. The quantification of *DENV-2 E* gene was normalized with *A. aegypti actin* (*AAEL011197*). The primers of dsRNA synthesis were shown in Table S3. All data were presented as mean ± standard error(SEM. The *Mann-Whitney* test was used for statistical analysis. The results were combined from 2 independent experiments.

Our previous study indicated that *mosGCTL-1* facilitated WNV infections, however, silencing *mosGCTL-1* did not influence DENV-2 infection in *A. aegypti* [9]. Given that *mosGCTL-1* belongs to a multi-gene family, we speculated that other *mosGCTL* paralogous, but not *mosGCTL-1*, may function as susceptibility factors in DENV infection of the mosquito. We therefore identified 36 genes encoding C-type lectin modules from the *A. aegypti* gene database (AaegL1.3); the database has been updated recently and contains more number of new gene transcripts than the previous version (https://www.vectorbase.org/organisms/aedes-aegypti) (Table S1). Double-stranded RNA (dsRNA)-mediated silencing in mosquitoes was then employed to assess the role of *mosGCTLs* in DENV-2 (New Guinea C strain) infection. Given the high sequence similarity among *mosGCTLs*, dsRNA for 33 of the 36 *mosGCTLs* were synthesized and individually microinjected into female *A. aegypti* mosquitoes. DENV-2 was sequentially inoculated 3 days later, and the effect on viral load was assessed 6 days after infection. Compared to the *GFP* dsRNA inoculated control, knockdown of 9 *mosGCTL* genes significantly reduced the DENV-2 burden in vectors (*p<0.05*) (Table S1 and Figure 1). We then determined the expression of *mosGCTL*s after gene silencing. The expression of 9 genes was reduced by 3 to 8-fold, compared with that in the controls (Figure S1), indicating that the impairment of dengue infection was correlated to *mosGCTLs* dsRNA inoculation.

The long dsRNA against one *mosGCTL* used in this study may potentially cross-react with another *mosGCTL* since *mosGCTL* family members share 30–70% nucleotide identity. We were therefore interested to know the specificity of dsRNA-mediated silencing among these *mosGCTLs*. The mRNA of 9 genes was measured using qPCR in mosquitoes treated with individual *mosGCTL* dsRNAs, and was then normalized with *A. aegypti actin*. Compared to the *GFP* dsRNA-inoculated control, *mosGCTL* genes were silenced with high efficacy and specificity. *mosGCTL-19*, *-22* and *-23* dsRNA cross-silenced several other family members (Table S2), indicating the phenotype of these 3 *mosGCTLs* may be influenced by dsRNA-mediated cross-silencing.

### *mosGCTL-3* facilitates DENV infection of *A. aegypti*

**Figure 2:**
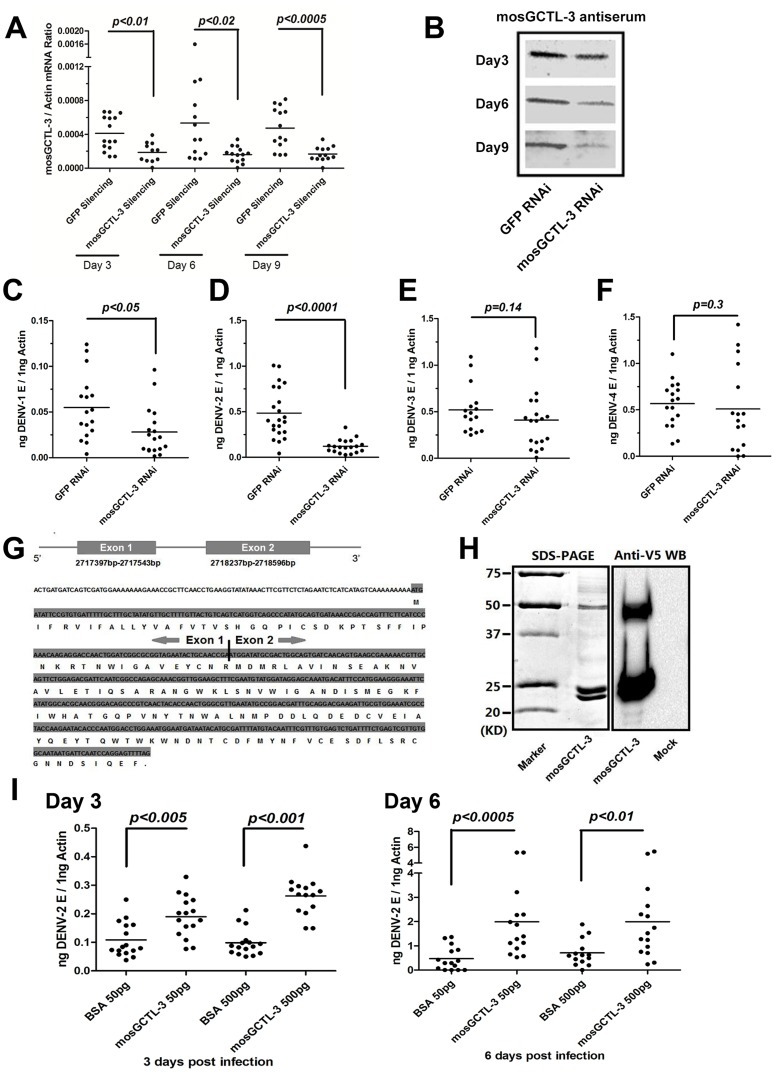
*mosGCTL-3* facilitated DENV infections. (**A–B**) Silencing efficiency of *mosGCTL-3* on both RNA level and protein level. 2 ug *GFP* or *mosGCTL-3* dsRNA was inoculated into mosquitoes thorax respectively. At 3, 6 and 9 days after the treatment, the mosquitoes were sacrificed to determine silencing effect by qPCR and normalized by *A. aegypti actin* (A). The primers of dsRNA synthesis and qPCR detection were described in Table S3. The *mosGCTL-3* silenced or mock mosquitoes were grinded in lysis buffer and mosGCTL-3 was detected by immunoblotting (B). (**C–F**) *mosGCTL-3* suppression impairs the infection of dengue serotypes. 10 M.I.D._50_ DENV-1 Hawaii strain (C), DENV-2 New Guinea C strain (D), DENV-3 Guangdong strain (E) and DENV-4 H241 strain (F) viruses were inoculated into *mosGCTL-3* silenced mosquitoes by microinjection respectively. The viral load was determined at 6 days post-infection by qPCR and normalized by *A. aegypti actin*. The result was pooled from 3 independent experiments. (**G**) Diagram of *mosGCTL-3* gene. An extra exon (Exon 1) of *mosGCTL-3*, which encoded a peptide with 20 amino acid signal sequence, was identified by the 5′-RACE. (**H**) Expression and purification of mosGCTL-3 by a *Drosophila* expression system. *mosGCTL-3* gene was cloned into pMT/BiP/V5-His-A DNA vector. The recombinant mosGCTL-3 was expressed and purified by a Cobalt-His column (Left panel). The expression was probed by anti-V5 mAb (Right panel). Mock was the concentrated supernatant of empty vector transfected *Drosophila* S2 cells. (**I**) Inoculation of mosGCTL-3 purified protein benefited DENV-2 infection in *A. aegypti*. 50 pg or 500 pg purified S2-expressed mosGCTL-3 protein was microinjected into each mosquito with 10 M.I.D._50_ DENV-2. The infected mosquitoes were sacrificed at 3 and 6 days post infection. The same amount of BSA was inoculated with DENV-2 as mocks. The viral load was determined by qPCR and normalized by *A. aegypti actin*. This experiment was repeated 3 times individually. One dot represented 1 mosquito and the horizontal line represented the mean value in all figures. We used the *Mann-Whitney* test for statistical analysis.

In the *in vivo* dsRNA-mediated screening, silencing *mosGCTL-3* (*AAEL000535*) led to the most significant reduction of DENV-2 burden and showed a high specificity. We therefore selected *mosGCTL-3* to evaluate the role of *mosGCTLs* in the infection of *A. aegypti* with DENV. The expression of *mosGCTL-3* was suppressed to study its role in the infection of 4 dengue serotypes. Total RNA from *GFP* (control) or *mosGCTL-3* dsRNA-injected mosquitoes was analyzed by reverse transcription-quantitative polymerase chain reaction (RT-QPCR). The expression of *mosGCTL-3* was decreased around 3-fold from 3 days through 9 days after dsRNA microinjection, compared to that in control mosquitoes (Figure 2A). The suppression was confirmed by immunostaining using mosquito lysate (Figure 2B). After 3 days of dsRNA treatment, 4 DENVs were separately inoculated into mosquitoes, and the viral burdens were quantified by qPCR at 6 days post infection. The burdens of DENV-2 (New Guinea C strain) were significantly reduced by *mosGCTL-3* silencing (*p<0.0001*, Figure 2D). Knockdown of *mosGCTL-3* showed a mild effect in DENV-1 (Hawaii strain) infection (*p<0.05*, Figure 2C). In contrast, suppression of the *mosGCTL-3* gene did not influence DENV-3 (Guangdong strain) or DENV-4 (H241 strain) loads in mosquitoes (Figure 2E and F).

Different strains of dengue may show varying levels of infectivity in mosquitoes. Except DENV-3 Guangdong strain [25], which is a low-passage strain isolated from patients, the other strains of three dengue serotypes are all laboratory-adapted high-passage viruses. To investigate whether *mosGCTL-3* plays a broader role in the replication of different dengue strains, we assessed the infectivity of multiple low-passage dengue strains. In accordance with observations of laboratory-adapted viruses, *mosGCTL-3* silencing significantly impaired the burdens of two low-passage DENV-2 strains (AF204178 and JX470186) (Figure S2B and C, *p<0.0001*). A mild effect was also observed in low-passage DENV-1 (FJ176780) infection (Figure S2A, *p<0.05*). However, knockdown of *mosGCTL-3* did not affect the replication of DENV-4 (JQ822247) in *A. aegypti* (Figure S2D), indicating the specificity of *mosGCTL-3* in the infection of various dengue serotypes.

C-type lectins are a family of proteins that recognize specific extracellular glycans. C-type lectins generally contain a signal peptide at their *N*-terminus that facilitates their C-type module exhibiting in the extracellular milieu. The predicted mRNA transcript of *mosGCTL-3* that was provided by the *A. aegypti* genome database, however, did not include any secretion signal. We speculated that an additional 5′ exon would be omitted, and therefore performed 5′-rapid amplification of cDNA ends (5′-RACE) to integrate the full length of *mosCGTL-3*. An extra exon of 147 bp, which encoded a peptide with a secretion signal of 20 amino acids (aa), was identified in the 5′ region of the gene (Figure 2G). To further understand the role of mosCGTL-3 in dengue infection, we expressed and purified mosGCTL-3 recombinant protein in a *Drosophila* S2 expression system (Figure 2H left panel). The mosGCTL-3 expression was probed by immunostaining with anti-V5 tag in recombinant protein (Figure 2H right panel). A previous study of the role of *mosGCTLs* in WNV infection demonstrated that mosGCTL proteins are soluble receptors in the extracellular milieu that enhance viral attachment and infection [9]. To elucidate the role of *mosGCTL-3* in dengue infection, we next pre-incubated purified S2-expressed mosGCTL-3 protein with DENV-2 and subsequently microinjected the combination into mosquito hemocoel; the inoculation of the same concentration of bovine serum albumin (BSA) with virus served as a negative control. At serial time points post-inoculation, the effects on DENV-2 burden was measured by qPCR. DENV-2 load was increased 2 to 4-fold in mosGCTL-3-treated mosquitoes compared with that in the controls on both day 3 and 6 (Figure 2I). The result was consistent with the prior results from the gene silencing experiments, directly indicating that the presence of mosGCTL-3 in the extracellular milieu enhanced dengue invasion in *A. aegypti*.

### Association of mosGCTL-3 and dengue infection

**Figure 3:**
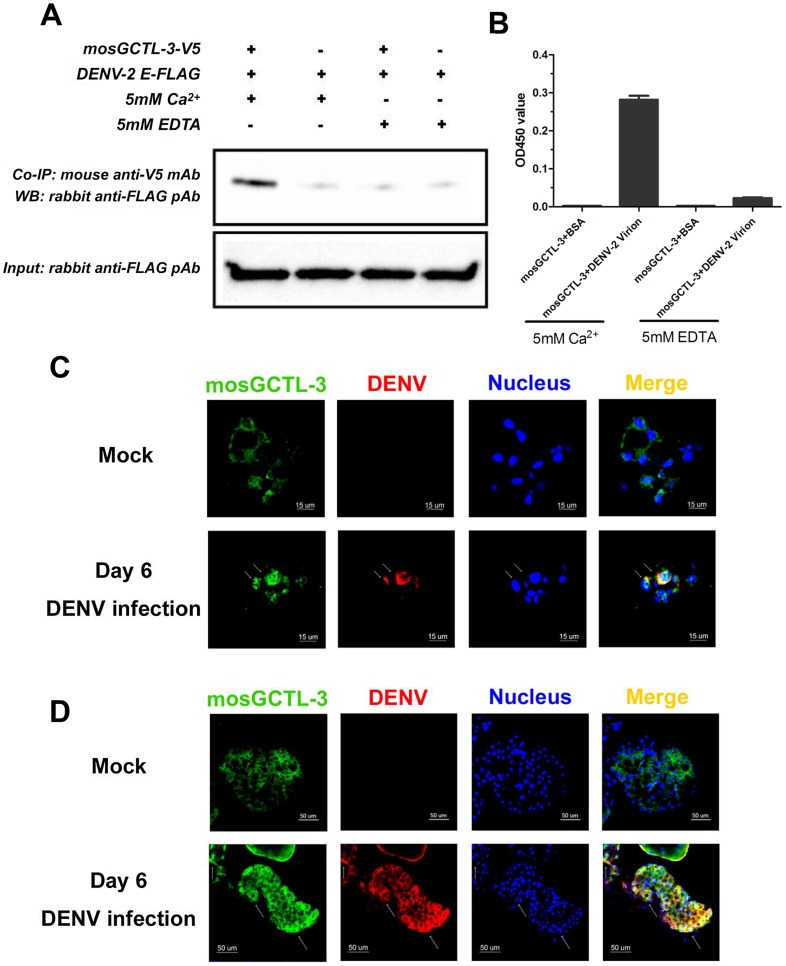
The interaction between mosGCTL-3 and DENV-2. (**A**) mosGCTL-3 interacted with DENV-2 Envelop protein in a co-immunoprecipitation (Co-IP) assay. The purified S2-expressed mosGCTL-3 and DENV-2 E proteins (2 ug each) were mixed for 2 h at 4°C. The protein complex was pulled down by anti-V5 mouse monoclonal antibody and probed by anti-FLAG rabbit polyclonal antibody. The experiment was repeated twice. (**B**) mosGCTL-3 captured DENV-2 virions by ELISA. The purified S2-expressed mosGCTL-3 was used in this experiment. The interaction was determined by a flavivirus E mAb 4G2. Data was expressed as mean ± standard error, and the experiment was reproduced three times. (**C–D)** The co-localization of mosGCTL-3 and DENV-2 in the tissues of *A. aegypti*. The mosquitoes were infected by intrathoracic microinjection. The salivary glands and hemocytes were then collected on 6 days after 1,000 M.I.D._50_ DENV-2 infection for immunofluorescence staining. The tissues of PBS inoculated mosquitoes were used as mocks. mosGCTL-3 was stained with anti-rabbit IgG Alexa-488 (Green), and DENV-2 E protein was probed by anti-mouse IgG Alexa-546 (Red). Nuclei were stained blue with To-Pro-3 iodide. The arrow represented the associated area between mosGCTL-3 and DENV-2 in hemocytes (C) and salivary glands (D). Images were examined by a Zeiss LSM 780 meta confocal with a Multi-Track mode.

C-type lectins recognize the glycans on the E protein of flaviviruses. Therefore, we investigated whether mosGCTL-3 interacts with dengue virus. As shown by co-immunoprecipitation (Co-IP) and ELISA, the purified S2-generated mosGCTL-3 directly binds to the DENV-2 E protein (Figure 3A) and virions (Figure 3B) in a calcium-dependent manner. To further examine the *in vivo* association, we generated mosGCTL-3 polyclonal antibodies for immunofluorescence staining in mosquito tissues. Co-localization of mosGCTL-3 and DENV-2 was clearly observed in mosquito salivary glands (Figure 3C) and hemocytes (Figure 3D). In the confocal microscopy studies, cells that were highly infected by dengue virus also strained positively for mosGCTL-3 in mosquito tissues. Our current results show that *mosGCTL-3* facilitated with DENV-1/2, but not DENV-3/4 infections, indicating the specificity of *mosGCTL* genes in the infectivity of dengue serotypes. To investigate whether the specificity correlated with interaction between mosGCTL-3 and DENVs, we used *Drosophila* S2 cells to generate the envelope (E) proteins of 4 dengue serotypes. An ELISA assay revealed that, compared to its weak interactions with DENV-3 and -4 E proteins, mosGCTL-3 (purified from S2 cells) showed the highest binding affinity for DENV-2 E, in addition to a mild affinity for DENV-1 E (Figure S3), indicating that DENV-ligand interaction may determine mosGCTL specificity of dengue serotypes infection.

The expression of *mosGCTL-3* in selected tissues of *A. aegypti* infected with DENV-2 was assessed. The virus was microinjected into the mosquito thorax and allowed to subsequently invade the tissues over time. The viral load in the hemolymph reached its peak at day 6; meanwhile, the infection levels increased in salivary glands from 3 to 12 days post-inoculation (Figure S4A). *mosGCTL-3* expression was substantially induced in the hemolymph and salivary glands at 3 and 6 days, and this was associated with a change in the viral burden in the tissue (Figure S4C and E). Mild induction of *mosGCTL-3* was also detected at an early stage of DENV-2 infection in the midgut and whole body (Figure S4B and D).

### Interruption of dengue infection by mosGCTL antisera

**Figure 4:**
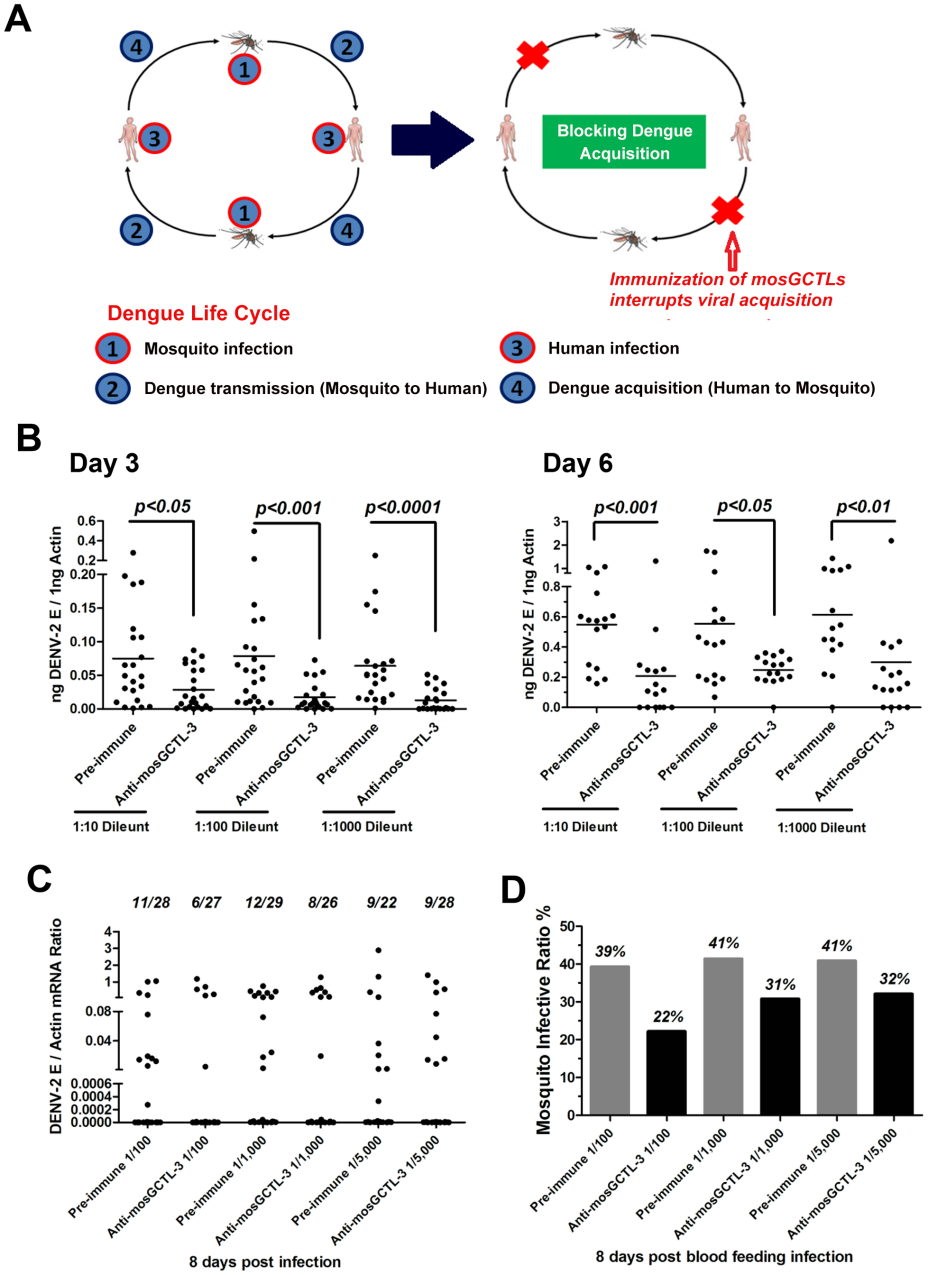
mosGCTL-3 antisera interrupted DENV-2 infection of *A. aegypti*. (**A**) Diagram of dengue life cycle and a transmission-blocking strategy for dengue prevention. (**B**) Inoculation of mosGCTL-3 antisera impaired DENV-2 infection of *A. aegypti*. The serial dilutions of mosGCTL-3 antisera or pre-immune sera with 10 M.I.D._50_ DENV-2 were inoculated into mosquitoes by microinjection. The infected mosquitoes were scarified at 3 days and 6 days post inoculation. DENV-2 was determined by qPCR and normalized by *A. aegypti actin*. One dot represented 1 mosquito and the horizontal line represented the mean value in all figures. The *Mann-Whitney* test was used for statistical analysis. The result was combined from 2 independent experiments. (**C–D**) mosGCTL-3 antisera interrupted DENV-2 infection by a blood meal. The mosGCTL-3 antisera or pre-immune sera were diluted 100-, 1,000- and 5,000-fold with fresh human blood and Vero cells-generated DENV-2. The mosquitoes were allowed to ingest the blood mixture by a membrane feeding. The fed mosquitoes were sacrificed 8 days later and DENV-2 infectivity was assessed by qPCR. The number of infected mosquitoes/total mosquitoes was presented on the top of each column (C). The mosquito infective ratio (D) was then interpreted from the Figure (C). One dot represented a mosquito. The result was pooled from 2 independent experiments.

Dengue virus is transmitted between the mosquito vector *A. aegypti* and human. The virus is transferred to the mosquito midgut while feeding on a viremic human. Subsequently, dengue virus overcomes midgut barriers and invades salivary glands, at which point the vector is ready for viral transmission. During the life cycle of dengue virus, viral transmission or acquisition may be interrupted to reduce the number of infected mosquitoes and facilitate dengue prevention (Figure 4A). Our current results showed that multiple mosGCTLs act as susceptibility factors for DENV-2 infection of mosquitoes (Figure 1). We therefore reasoned that immunization of the host against mosGCTLs may impair dengue infection during a blood meal (Figure 4A). Among the identified mosGCTLs in DENV-2 infection, mosGCTL-3 had the greatest association with susceptibility. Therefore, we first generated mosGCTL-3 antisera using *Escherichia coli*-expressed recombinant antigen in rabbits and then validated the antisera by immunoblotting. The antisera recognized mosGCTL-3 generated from *Drosophila* cells (Figure S5A) and the native protein in mosquito lysate (Figure S5B). To determine the effects of mosGCTL-3 antisera on dengue infection, we first microinjected serial dilutions of mosGCTL-3 antisera and DENV-2 (New Guinea C strain) into mosquitoes. Compared to the control mosquitoes that were treated with pre-immune sera, dengue burden was reduced 2 to 4-fold in mosquitoes that received mosGCTL-3 antisera at day 3 and 6 (Figure 4B), indicating that blocking mosGCTL-3 efficiently impaired dengue infection in *A. aegypti*. We next examined whether mosGCTL-3 antisera interrupted dengue infection by membrane feeding. Mosquitoes engorged on 1∶100, 1∶1,000, and 1∶5,000 dilutions of mosGCTL-3 antisera, mixed with DENV-2 (New Guinea C strain) and fresh human blood. The same dilutions of pre-immune sera served as controls. After 8 days of rearing in standard condition, the fed mosquitoes were sacrificed and DENV-2 infectivity was assessed by qPCR. Compared to the 39–41% infective ratio for mosquitoes fed with pre-immune sera, the infective ratio was decreased to 22–32% in mosquitoes treated with mosGCTL-3 antisera. A higher concentration (1∶100 dilution) of mosGCTL-3 antisera resulted in greater levels of inhibitory activity against DENV-2 infection (Figure 4C and D).

**Figure 5:**
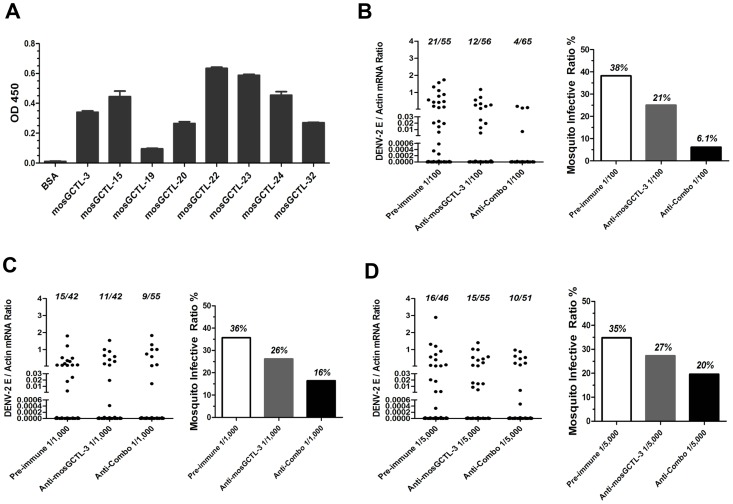
Feeding the combination of 9 mosGCTLs antisera blocked DENV-2 uptake by a membrane blood feeding. (**A**) The interaction of the susceptible mosGCTLs and DENV-2 E protein. The *mosGCTL* genes exhibiting susceptible role in DENV-2 infection (*p<0.05*) were cloned into pMT/Bip/V5-His A DNA vector. The recombinant mosGCTLs with a V5 tag in C-terminus were expressed in *Drosophila* S2 cells. mosGCTL-26 (*AAEL017265*) was failed to be expressed in *Drosophila* S2 cells. The interaction was determined by ELISA. An anti-V5 mouse mAb was used to probe the binding activity. The experiment was reproduced twice. (**B–D**) Feeding the combination of 9 mosGCTL antisera blocked DENV-2 infection. The pre-immune sera, mosGCTL-3 antisera and combined mosGCTLs antisera (1/9 mosGCTL antiserum each) were serially diluted 100- (B), 1,000- (C), or 5,000- (D) fold with fresh human blood and Vero cells-generated DENV-2. The fed mosquitoes were sacrificed on 8 days post infection to determine the infectivity by qPCR. The number of infected mosquitoes/total mosquitoes was presented on the top of each column (Left panel of each subfigure). The mosquito infective ratio (Right panel) was then interpreted from the left panel. One dot represented a mosquito. The result was pooled from 4 independent experiments.

Functional screening showed that silencing the paralogous of 9 *mosGCTLs* decreased DENV-2 burden in *A. aegypti* (Figure 1), implying that the virus employs multiple *mosGCTLs* to enhance the infection. We next cloned the other 8 *mosGCTLs*, expressed the recombinant proteins in *Drosophila* S2 cells, and determined the interaction between these mosGCTLs and DENV-2. Except for mosGCTL-26 (*AAEL017265*), which failed to be expressed in S2 cells, all 7 mosGCTL recombinant proteins were shown to bind to DENV-2 E protein by ELISA (Figure 5A). We therefore produced antisera for the other 8 identified mosGCTLs by immunization and validated the antisera by immunostaining (Figure S5A and B). The combination of 9 diluted mosGCTL antisera (antisera combo) was mixed with fresh human blood and DENV-2 (New Guinea C strain) for a membrane blood meal. Mosquitoes were sacrificed to determine the infective ratio at 8 days post-feeding. In the 1∶100 dilution, infective ratio was 38% for the mosquitoes fed pre-immune sera, 21% for those fed mosGCTL-3 antisera, however only 6% for those fed the antisera combo (Figure 5B). The mosquito infective ratio was found to be correlated with the fed amount of antisera against mosGCTLs in a dose-dependent manner (Figure 5B–D).

**Figure 6:**
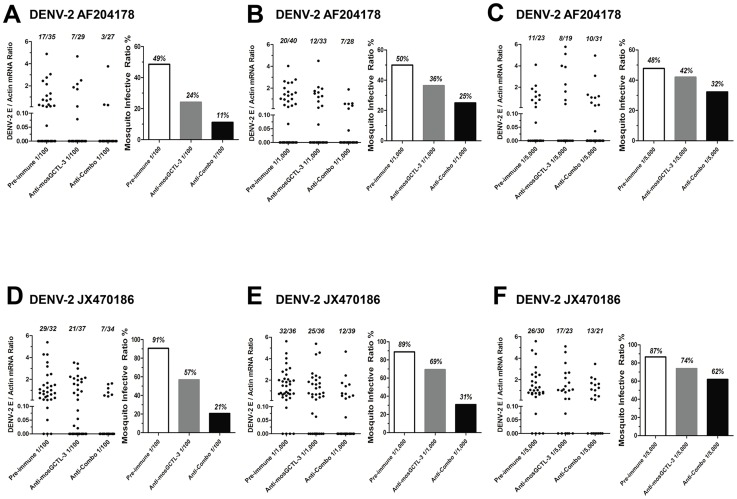
Transmission-blocking effect of mosGCTLs antisera in the infection of low-passage DENV-2 strains. (**A–F**) Two low-passage DENV-2 strains, which were isolated from patients' sera, were employed to test transmission-blocking effect of mosGCTL antisera in *A. aegypti*. The pre-immune sera, mosGCTL-3 antisera and combined mosGCTLs antisera (1/9 mosGCTL antiserum each) with Vero cells-generatedDENV-2 AF2014178 strain (A to C)/JX470186 strain (D to F) and human blood were used for mosquitoes feeding. The antisera were serially diluted 100- (A and D), 1,000- (B and E) and 5,000- (C and F) fold for the investigation. The fed mosquitoes were sacrificed on 8 days post infection to determine the infectivity by qPCR. The numbers of infected mosquitoes/total mosquitoes are presented on the top of each column (Left panel of each sub-figure). The mosquito infective ratio (Right panel) indicates the ratio of DENV positive to total mosquitoes. One dot represents a mosquito. The results are pooled from 3 independent experiments.

Upon dsRNA-mediated *mosGCTL-3* silencing, infection of *A. aegypti* with the two low-passage DENV-2 strains was significantly reduced (Figure S2B and C). We next determined the transmission-blocking effect of mosGCTLs antisera in infection with low-passage DENV-2 strains (AF204178 and JX470186). For the mosquitoes fed with DENV-2 AF204178 virus, the infective ratio was 49% in 1∶100 diluted pre-immune sera group, 24% in 1∶100 diluted mosGCTL-3 antisera group, however, only 11% of mosquitoes became infected after being fed with 1∶100 diluted antisera combo (Figure 6A). Likewise, in DENV-2 JX470186 infection, in the 1∶100 dilution, compared to 91% infective ratio for mosquitoes fed with pre-immune sera and 57% for those fed with mosGCTL-3 antisera, the infective ratio was decreased to 21% in the group of antisera combo (Figure 6D). Meanwhile, the inhibitory effect was reduced with the dilution of the fed amount of mosGCTLs antisera (Figure 6A–F), indicating the correlation of infective ratio and uptake of mosGCTL antisera by mosquitoes. Hence, blocking mosGCTLs function with antibodies might be a feasible approach for interrupting dengue infection, and human immunization against mosGCTLs shows promise as a strategy for developing a vaccine for dengue prevention.

## Discussion

C-type lectins are a group of evolutionarily conserved proteins with sugar binding activity. Several C-type lectins were identified as ligands interacting with arboviruses in the infection of both hosts and vectors. In humans, multiple C-type lectins recognize glycans on the surface of arboviruses to facilitate viral entry or contribute to inflammation-related pathogenesis [26], [27], [28], [29], [30]. In vectors, a mosquito C-type lectin, designated as mosGCTL-1, is a receptor that cooperates with a membrane-bound protein (mosPTP-1) to enhance WNV infection [9]. DENV is a flavivirus and similar to WNV in its genetic structure. We reasoned that mosGCTLs may also recognize DENV and play a role in DENV infections. Therefore, we screened the *mosGCTL* family by dsRNA-mediated *in vivo* silencing and identified 9 genes that facilitate DENV-2 invasion. Further results showed that the identified functional mosGCTLs interacted with DENV-2 E protein efficiently, indicating that DENV recruited multiple mosGCTLs for the infection of vectors. As the primary vectors for the transmission of many arboviruses, mosquitoes may have developed a general mechanism to facilitate microbial invasion. mosGCTLs are vector ligands that directly interact with viral surface proteins to enhance infection. Therefore, we speculated that mosGCTLs may play a broad role in expediting many viral infections; this may not be limited to WNV and DENV, but may extend to other arboviruses. Further, our studies have indicated that WNV and DENV employ different mosGCTLs to promote infection. Considering these results, we hypothesize that (1) mosGCTLs may function as general susceptibility factors for arboviral infections of mosquitoes, (2) Different mosGCTLs may exhibit specificity for different arboviruses, and (3) Immunization of the mammalian host with mosGCTLs could potentially reduce transmission of a broad spectrum of arboviruses.

The binding affinity between mosGCTL-3 and DENV E proteins may determine *mosGCTL* specificity in dengue serotypes infection. Based on the common property of C-type lectins, we speculate that mosGCTLs may recognize the glycans of dengue E proteins that are similar among four serotypes. However, our results clearly show that mosGCTL-3 has differential binding affinity for different dengue E proteins. To explain the binding variations between mosGCTL-3 and DENV E proteins, we investigated the surface residues nearby the glycosylation sites that may affect the interaction. Analyses of the structure of DENV-2 in complex with the carbohydrate recognition domain (CRD) of DC-SIGN showed that one CRD monomer bound to two glycosylation sites at Asn67 of two neighboring E proteins [31]. In light of the structural complexity, the protruding surface residues within the interaction interface between the CRD and the virus may affect the binding affinity of the CRD for the virus. In the case of DC-SIGN CRD, five residues that are close to Asn67 and are within the interaction interface could be identified, including Thr66/Glu84/Arg89/Met118/Thr120 (PDB accession number: 2b6b). Alignment of sequences of 4 dengue E proteins showed that these protruding surface residues vary greatly among the four different dengue serotypes (data not shown). The CRD of DC-SIGN does not recognize Asn153, which is the other glycosylation site on the DENV-2 E protein [31]; however, mosGCTL CRD may recognize this site. We compared the protruding surface residues near Asn153 and found that these nearby residues (Glu147/Glu148/His149/Thr155/Lys157) are also not conserved among the 4 dengue E proteins (data not shown); this may potentially influence the strength and affinity of mosGCTL-3 binding to DENV virions. Furthermore, a previous study showed that DC-SIGN-transfected THP-1 cells exhibited marked differences in infectivity among dengue serotypes [32]. Based on our data and previously published results, we hypothesize that the differing properties of the protruding surface residues near glycosylation sites of dengue E proteins may determine the binding affinity of mosGCTLs for dengue E proteins.

As soluble C-type lectins in the plasma, mosGCTLs are deemed to be homologues of the human mannose binding lectin (MBL). MBL is a well-known innate immune factor for recognizing microbial invasion and activating downstream complement cascades. MBL also interacts with several membrane ligands to induce immune stimulation. Human CD45 is a protein tyrosine phosphatase receptor that is important for T cell activation and is expressed on all nucleated cells of hematopoietic origin [33], [34], [35]. CD45 can directly interact with MBL to influence thymocyte development [36]. mosPTP-1 was identified as a membrane-bound protein tyrosine phosphatase (PTP) receptor that shares homology with human CD45. In WNV infections, mosGCTL-1 and mosPTP-1 enable the attachment of the WNV on the cell surface, thereby enhancing viral entry [9]. In the current study, we assessed the role of *mosPTP-1* in DENV-2 infection; however, silencing *mosPTP-1* did not influence DENV-2 burden in *A. aegypti* (data not shown). Bioinformatic analysis revealed that *mosPTP-1* belongs to a multi-gene family with the PTP domain. We therefore speculate that, similar to the specificity of mosGCTLs for different flaviviruses, dengue virus may employ another *mosPTP* paralogue to mediate viral entry into mosquito cells.

For vector-borne diseases, the stages of the life cycles of pathogen can be targeted for vaccine design to enable the control of the diseases in the environment. A transmission-blocking vaccine targeting the gametocytes of the malaria pathogen, *Plasmodium spp.*, successfully reduces the number of parasites surviving in the mosquito gut and consequently impairs the efficiency of microbial acquisition and mosquito infectivity [37], [38]. Several surface antigens expressed during the sexual stage of *Plasmodium*, such as Pfs 48/45 and Pfs 25/28, were selected as the targets for the vaccine. The immunity elicited by the vaccination efficiently blocks the uptake of *Plasmodium* by the mosquito vector [37], [38]. Along with antigens of particular importance in the life cycles of parasites, the vector ligands that interact with pathogens are other candidates for vaccine development. An aminopeptidase N (AgAPN1) was characterized as a conserved putative ligand for both murine and human *Plasmodium* ookinetes in diverse *Anopheles spp.*. The polyclonal antibodies against AgAPN1 strongly inhibited *Plasmodium* development in mosquito midgut [39], [40]. Humoral immunity against mosGCTL-1 has been shown to significantly reduce vectorial capacity for WNV infection during a blood meal [9]. Host humoral immunity against TROSPA, a tick gut receptor for the Lyme disease agent, limits the colonization of ticks by *B. burgdorferi* [24]. Therefore, strategies that target the life cycle of vector-borne microbes are feasible approaches to reducing the global diseases burden.

There are no licensed dengue vaccines or therapeutics available. A promising live-attenuated tetravalent dengue vaccine developed by Sanofi Pasteur has been shown to induce partial protection against infection with dengue virus serotypes and is being advanced in clinical trials [41]. Given the rapid increase in dengue spread and disease burden over the last decade, additional strategies are urgently needed to combat dengue dissemination [42], [43], [44], [45]. The survival of the dengue virus is restricted to its interaction between humans and *Aedes* mosquitoes. The host specificity in dengue infection implies that vaccination of the human population is feasible as a means to reduce the number of infected vectors and consequentially reduce disease burden. Here, we identified that multiple mosGCTLs interacted with DENV-2 virus to enhance viral infection in *A. aegypti*. Compared to treatment with pre-immune sera or mosGCTL-3 antiserum alone, the combination of antisera against multiple mosGCTLs dramatically reduced dengue infection after a membrane blood meal, indicating that mosGCTL immunization in human may help in interrupting the life cycle of the dengue virus in nature. However, the host specificity is a major obstacle to testing our vaccine strategy in an *in vivo* system. Human is the only susceptible vertebrate host, developing high viremia after dengue infection (urban epidemic strains) that allows mosquitoes to acquire the infection during blood feeding. Therefore, most of the commonly used animal models, in which the viral load in blood is not sufficient to infect mosquitoes, are not suitable for the investigation of dengue acquisition. Several transgenic mouse lines and non-human primates (NHPs) are susceptible to dengue infection and have been widely used as models for understanding dengue pathogenesis and drug discovery [46]. We will test the feasibility of which the mosquitoes acquire dengue infection with these models. Furthermore, we plan to perform passive and active immunization against mosGCTLs in a suitable animal model and assess the efficacy of our vaccine strategy.

A major concern regarding the feasibility of the mosGCTL-based transmission blocking strategy is the concentration and persistence of mosGCTL antibodies after human immunization. As shown in a study on Enterovirus 71 (EV71) vaccine for humans, titers of neutralizing antibodies may decline only slowly and persist years after the final immunization [47]. Moreover, the titers of WNV-neutralizing antibodies remained high and did not decrease for more than one year after acute West Nile virus infection in humans [48]. Our results indicate that oral feeding of 1∶100 dilution of rabbit mosGCTLs antisera, generated from 3 booster immunizations, efficiently interrupted DENV-2 infection in *A. aegypti*. A 1∶1,000 dilution of anti-mosGCTL antisera also achieved an effect. In nature, mosquitoes ingest undiluted human whole blood. The promising transmission-blocking effects of diluted anti-mosGCTL antisera support that the titers of antibodies elicited by mosGCTLs immunization in human may be sufficient to interrupt the life cycle of the dengue virus over a long period and to control the disease burden in nature. Another concern for the vaccine strategy is that the efficacy of simultaneous immunization with multiple antigens would be complicated by the differential immunogenicity of each antigen. Future research efforts should focus on improving the efficacy of immunization with multiple antigens. One feasible approach is to design common antigenic motifs in all mosGCTL proteins using structural and bioinformatics tools.

In this study, we identified multiple *mosGCTLs* that facilitate dengue infection; these mosGCTLs directly interacted with DENV-2 *in vitro* and *in vivo*. The combination of antisera against multiple mosGCTLs efficiently reduced DENV-2 infection after a blood meal, suggesting that it is feasible to develop a mosGCTL-based transmission-blocking vaccine against dengue dissemination. Given the binding activity of C-type lectins and viral glycans, we reasoned that mosGCTLs may function as key susceptibility factors for many arboviral pathogens. In future studies, we plan to determine the role of *mosGCTLs* in other mosquito-borne viral infections and to develop sophisticated mosGCTL-based vaccination strategies against the transmission of multiple mosquito-borne viruses in nature. This study substantially extends our understanding of flaviviral replication in vectors and provides a research avenue by which the development of therapeutics for preventing the dissemination of mosquito-borne viral diseases can be pursued in the future.

## Materials and Methods

### Ethics statement

Collection of human blood samples was conducted with approval of the local ethics committee at Tsinghua University. Human blood taken from healthy donors, who provided written informed consent, was used for mosquito blood feeding.

### Mosquitoes, cells, and viruses

The Rockefeller strain of *A. aegypti* mosquitoes were maintained in an incubator (Precision 3758CN; Thermo Scientific) at 28°C and 80% humidity according to standard rearing procedures. DENV-1 Hawaii, DENV-1 FJ176780 [49], DENV-2 New Guinea C, DENV-2 AF204178 [50], DENV-2 JX470186 [51], DENV-3 Guangdong [25], DENV-4 H241 and DENV-4 JQ822247 [52] strains were grown in *A. albopictus* C6/36 cells for intrathoracic inoculation, and three DENV-2 strains (New Guinea C; AF204178; JX470186) were amplified in Vero cells for blood meals [9]. Both C6/36 and Vero cell lines were maintained in Dulbecco's Modified Eagles Medium supplemented with 10% heat-inactivated fetal bovine serum (16000-044; Gibco) for DENVs production. The *Drosophila melanogaster* S2 cell line was cultured in Schneider's Medium with 10% heat-inactivated fetal bovine serum (Gibco). DENVs were titrated by both plaque formation assay and 50% mosquito infectious dose (M.I.D_50_) as described previously [9], [53], [54].

### Antibodies and antisera generation

A flaviviral E protein 4G2 monoclonal antibody was produced from a hybridoma cell line (D1-4G2-4-15; ATCC). The antibodies for tags were purchased from Medical & Biological Laboratory (MBL, Japan). For antigens production, *mosGCTL* genes without the predicted signal sequences were amplified from *A. aegypti* cDNA and cloned into pET28a(+) expression vector. The cloning primers are presented in the Table S3. The recombinant mosGCTL proteins were expressed in *Escherichia coli* BL21 DE3 strain, with insoluble form in inclusion bodies. The proteins were then resolved by 8 M urea and purified by TALON purification Kit (635515; Clontech). The polyclonal antibodies were produced by immunizing rabbits with recombinant mosGCTLs, including 3 boosting immunizations.

### Intrathoracic microinjection

For investigating the gene functions, materials including dsRNA, purified proteins, antibodies, and dengue viruses were inoculated into the thorax of *A. aegypti*. The microinjection procedure was introduced and described in our previous study [9]. Briefly, female *A. aegypti* mosquitoes were placed on a cold tray cold-anaesthetised, and materials were injected into their thoraxes. The injected mosquitoes were further reared in the standard condition for following experiment or detection.

### 5′-Rapid Amplification of cDNA Ends (5′-RACE)

The 5′ terminus of *mosGCTL-3* was amplified using a 5′-Full RACE Kit (D315; TaKaRa, Japan). Total RNA of female *A. aegypti* was isolated by an RNeasy Mini Kit (74106; Qiagen). The specific outer and inner primers of *mosGCTL-3* were described in Table S3. The experimental details of were described in the product manual of 5′-Full RACE Kit. The amplified fragments was subcloned into pMD-18T Simple Vector (D103A; TaKaRa) for sequencing.

### Protein generation in a *Drosophila* expression system

The *mosGCTL* genes without signal sequences were cloned into pMT/BiP/V5-His A (V4130-20; Invitrogen) for expression in S2 cells. The primers were shown in Table S3. The generation of stable cells was described in the product manual of the *Drosophila* expression system (K5130-01; Invitrogen). The stable cell lines were amplified in regular medium in a 175 cm^2^ flask and then transferred into spinner flasks with serum-free medium (10486-025; Gibco) for protein expression. The cells were cultured for 3 days and induced with 500 µM copper sulfate for 4 days. The supernatant was centrifuged, filtrated, and then concentrated for purification with a Talon metal affinity resin (635515; Clontech). The protein purity was checked by SDS-PAGE and immunostaining with an anti-V5 mouse monoclonal antibody (M167-3; MBL, Japan).

### Quantitative-Polymerase Chain Reaction (qPCR)

The cDNAs of DENV genome were synthesized using a cDNA reverse transcription kit (170-8890; Bio-Rad) and quantified by qPCR with specific probes. The expression of *mosGCTL* genes was measured by qPCR with SYBR supermix (170-8880; Bio-Rad). The primers and probes were shown in Table S3. Gene quantities were normalized using *A. aegypti* actin (*AAEL011197*).

### Co-immunoprecipitation

The purified S2-expressed mosGCTL-3 and DENV-2 E proteins (2 ug each) were mixed for 2 h at 4°C. We pulled down mosGCTL-3, and DENV-2 E was detected using an anti-FLAG antibody (PM020; MBL, Japan). The experimental details were described in the product manual of the commercial IP kit (26146; Thermo Scientific).

### ELISA

The microtitre test plate (Nunc, Denmark) was coated with 2 µg purified DENV-2 E protein overnight at 4°C. After 3 washings with phosphate buffered saline (PBS) containing 0.05% Tween 20 (PBST), the plates were blocked using 5% w/v BSA solution for 1 h at room temperature (RT). After 5 washings, recombinant mosGCTLs were added to each well and incubated at RT for 2 h. The wells were then washed 5 times with PBST. Primary antibody was added, and incubation continued at RT for 2 h. After washing again, 100 µL of secondary IgG-HRP was added to each well, and the plates were incubated at RT for 1 hr. The commercial peroxidase substrate system was used for signaling detection (52-00-01 and 50-85-04; Kirkegaard & Perry Laboratories, USA), and the optical density (OD) at 450 nm was measured with an ELISA reader.

The interaction between mosGCTL-3 and DENV-2 virions was also measured using ELISA. In the procedure, the microtitre test plate was coated with 2 µg purified S2-expressed mosGCTL-3 protein at 4°C overnight. After 3 washings with PBST, the plates were blocked using 5% w/v BSA solution for 1 h at room temperature. After 5 washings, 2 µg purified inactivated DENV-2 virions (EL-22-02-001; MicroBix, Canada) in PBS was added to each well and incubated for 2 h at 4°C. After washing with PBST, a flavivirus E protein 4G2 mAb was added, and further incubated for 2 h at 4°C. The analysis followed the procedure outlined above.

### Imaging of mosquito tissues

*A. aegypti* salivary glands and hemocytes were dissected and stained as previously described [9], [11], [55]. Tissues were dissected and placed on sialylated slides (PGC Scientific, USA) and fixed in 4% paraformaldehyde plus 0.1% Triton X-100 at RT for 1 hr. After staining for primary and secondary antibodies, the slides were imaged using a Zeiss LSM 780 meta confocal microscope (Carl Zeiss, Germany) with a Multi-Track mode.

### Membrane blood feeding

Fresh human blood was collected in heparin tubes (367884; BD Vacutainer) and centrifuged at 3,000 rpm for 10 min to separate the plasma from the blood cells. The plasma was collected and heat-inactivated at 55°C for 30 min. The blood cells were washed 3 times in PBS to remove the anticoagulant. The washed cells were then suspended with the treated plasma. Antisera against mosGCTLs or pre-immune sera were mixed with DENV-2 virus and human blood to feed mosquitoes using the blood feeding system (6W1; Hemotek Limited, England). The mosquitoes were anesthetized at 4°C. Subsequently, the fed female mosquitoes were identified and transferred into new containers and maintained with 1% sucrose solution for 8 days. The mosquitoes were sacrificed and homogenized for total RNA isolation (74106; Qiagen). DENV-2 genome was reversely transcripted and quantified by qPCR assay.

## Supporting Information

Figure S1 **dsRNA-mediated silencing efficiency of** ***mosGCTL*** **genes in** ***A. aegypti*** **.** (**A–I**) *mosGCTLs* dsRNA were inoculated into mosquitoes respectively. *GFP* dsRNA served as mock control. The mosquitoes were sacrificed at 9 days after dsRNA inoculation. The expression of *mosGCTL* genes was determined by qPCR and normalized by *A. aegypti actin*. The qPCR primers were shown in Table S3. One dot represented 1 mosquito and the horizontal line was the mean value in all figures. The Mann-Whitney test was used for statistical analysis.
(PDF)

Figure S2 **Determine the effect of** ***mosGCTL-3*** **silencing in the infection of multiple low-passage DENV strains.** (**A–D**) 10 M.I.D._50_ DENV-1 FJ176780 (A), DENV-2 AF204178 (B), DENV-2 JX470186 (C) and DENV-4 JQ822247 (D) viruses were inoculated into *mosGCTL-3* silenced mosquitoes by microinjection respectively. The viral load was determined at 6 days post infection by qPCR and normalized by *A. aegypti actin*. The primers and probes were shown in Table S3. One dot represented 1 mosquito and the horizontal line was the mean value in all figures. The Mann-Whitney test was used for statistical analysis. The result was pooled from 2 independent experiments.
(PDF)

Figure S3 **The interaction between purified mosGCTL-3 and the E proteins of 4 dengue serotypes by ELISA.** (**A**) The 4 DENV Envelope genes (DENV-1, Hawaii; DENV-2, New Guinea C; DENV-3, Guangdong; DENV-4, H241) with a FLAG tag were cloned into pMT/Bip/V5-His A DNA vector. The recombinant DENV E proteins were expressed in *Drosophila* S2 cells. The interaction was determined by ELISA. In the study, the purified mosGCTL-3 (2 ug) was coated on the wells. BSA served as a mock control. The S2 expressed DENV E proteins was then respectively incubated in the wells (the amount balanced by Western-blotting shown in Figure S3B). An anti-FLAG mouse mAb was used to probe the interaction. The experiment was reproduced three times with the similar results. (**B**) Determination of the loading E proteins in ELISA. The DENV E proteins expressed in S2 cells were measured by Western-blotting with anti-FLAG mAb.
(PDF)

Figure S4 **The expression of** ***mosGCTL-3*** **during DENV-2 infection of** ***A. aegypti*** **tissues.** (**A**) DENV-2 distribution in selected *A. aegypti* tissues. The virus was inoculated into mosquito thorax by microinjection and DENV-2 load was determined by qPCR and normalized by *A. aegypti actin*. (**B–E**) The regulation of *mosGCTL-3* by DENV-2 infection in mosquito tissues, including whole body (B), salivary glands (C), midgut (D), and hemolymph (E). Total RNA was isolated from various tissues or whole mosquitoes at time courses. Each group included at least 9 individual tissues or mosquitoes.
(PDF)

Figure S5 **Validation of mosGCTLs polyclonal antibodies.** (**A–B**) mosGCTLs antisera were produced by immunization of the *E.coli*-expressed antigens in rabbits. The mosGCTLs polyclonal antibodies were validated by detection of the recombinant mosGCTLs proteins from S2 cells (A) and the native proteins in mosquito lysate (B).
(PDF)

Table S1 **The role of 36** ***mosGCTL*** **genes in DENV-2 infection in** ***A. aegypti*** **.** Double-stranded RNAs (dsRNA) against *mosGCTLs* were synthesized and microinjected into mosquito thorax to knock down the target gene. After 3 days post dsRNA microinjection, 10 M.I.D_50_ (Mosquito Infective Dose 50%) DENV-2 was injected into the mosquitoes. After 6 days, mosquitoes were sacrificed and the virus burden assessed. 9 of the 33 genes showed a significant decrease of the virus burden (*p<0.05*). Statistical analysis was done with the *Mann-Whitney* test.
(PDF)

Table S2 **The specificity of the dsRNA-mediated silencing among the 9** ***mosGCTL*** **genes.** The number represents the ratio of the *mosGCTL* mRNA amount between *mosGCTL*-dsRNA and *GFP*-dsRNA treated mosquitoes. The *mosGCTLs* dsRNA were inoculated into mosquitoes respectively. *GFP* dsRNA served as mock control. The mosquitoes were sacrificed at 9 days after dsRNA inoculation. The amount of *mosGCTL* mRNA was determined by qPCR and normalized by A. aegypti actin.
(PDF)

Table S3 **Primers and probes for qPCR, dsRNA synthesis and gene cloning.**
(PDF)
