# Supplementary material for: Transmission-Blocking Antibodies against Mosquito C-Type Lectins for Dengue Prevention
Source: PLoS Pathog. 2014 Feb 13;10(2):e1003931. doi: 10.1371/journal.ppat.1003931 (PMC3923773; doi:10.1371/journal.ppat.1003931)
Supplement: Figure S1 — dsRNA-mediated silencing efficiency of mosGCTL genes in A. aegypti . (A–I) mosGCTLs dsRNA were inoculated into mosquitoes respectively. GFP dsRNA served as mock control. The mosquitoes were sacrificed at 9 days after dsRNA inoculation. The expression of mosGCTL genes was determined by qPCR and normalized by A. aegypti actin. The qPCR primers were shown in Table S3. One dot represented 1 mosquito and the horizontal line was the mean value in all figures. The Mann-Whitney test was used for statistical analysis. (PDF) [file ppat.1003931.s001.pdf]

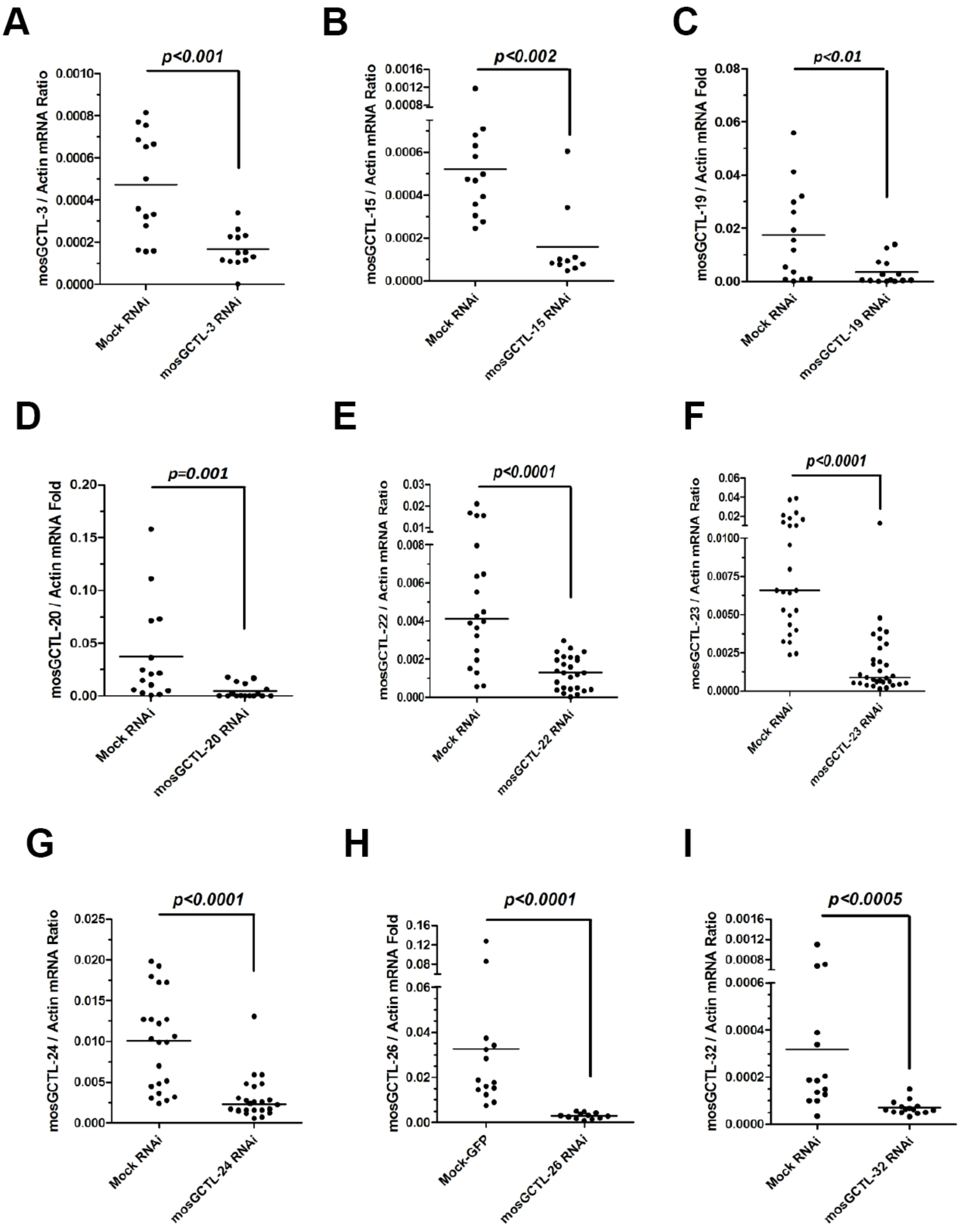

Figure S1. dsRNA-mediated silencing efficiency of *mosGCTL* genes in *A. aegypti*.

(**A-I**) *mosGCTLs* dsRNA were inoculated into mosquitoes respectively. *GFP* dsRNA served as mock control. The mosquitoes were sacrificed at 9 days after dsRNA inoculation. The expression of *mosGCTL* genes was determined by qPCR and normalized by *A. aegypti actin*. The qPCR primers were shown in Table S3. One dot represented 1 mosquito and the horizontal line was the mean value in all figures. The Mann-Whitney test was used for statistical analysis.
