# Supplementary material for: Transmission-Blocking Antibodies against Mosquito C-Type Lectins for Dengue Prevention
Source: PLoS Pathog. 2014 Feb 13;10(2):e1003931. doi: 10.1371/journal.ppat.1003931 (PMC3923773; doi:10.1371/journal.ppat.1003931)
Supplement: Figure S2 — Determine the effect of mosGCTL-3 silencing in the infection of multiple low-passage DENV strains. (A–D) 10 M.I.D.50 DENV-1 FJ176780 (A), DENV-2 AF204178 (B), DENV-2 JX470186 (C) and DENV-4 JQ822247 (D) viruses were inoculated into mosGCTL-3 silenced mosquitoes by microinjection respectively. The viral load was determined at 6 days post infection by qPCR and normalized by A. aegypti actin. The primers and probes were shown in Table S3. One dot represented 1 mosquito and the horizontal line was the mean value in all figures. The Mann-Whitney test was used for statistical analysis. The result was pooled from 2 independent experiments. (PDF) [file ppat.1003931.s002.pdf]

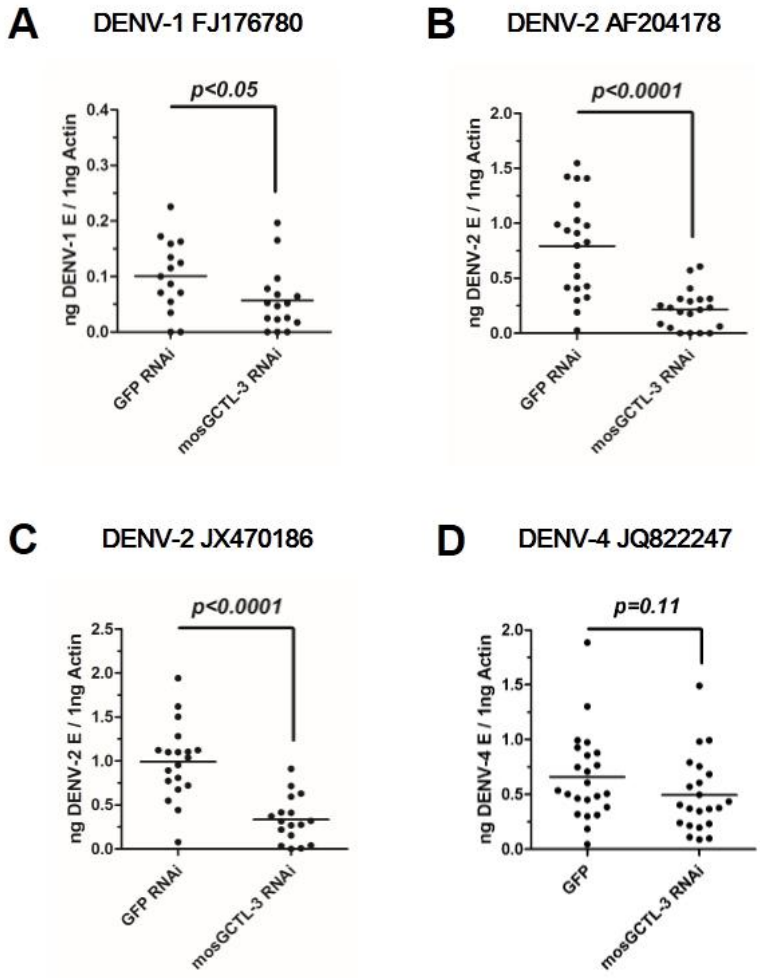

Figure S2. Determine the effect of *mosGCTL-3* silencing in the infection of multiple low-passage DENV strains.

(**A-D**) 10 M.I.D.$_{50}$ DENV-1 FJ176780 (A), DENV-2 AF204178 (B), DENV-2 JX470186 (C) and DENV-4 JQ822247 (D) viruses were inoculated into *mosGCTL-3* silenced mosquitoes by microinjection respectively. The viral load was determined at 6 days post infection by qPCR and normalized by *A. aegypti actin*. The primers and probes were shown in Table S3. One dot represented 1 mosquito and the horizontal line was the mean value in all figures. The *Mann-Whitney* test was used for statistical analysis. The result was pooled from 2 independent experiments.
