# Supplementary material for: Transmission-Blocking Antibodies against Mosquito C-Type Lectins for Dengue Prevention
Source: PLoS Pathog. 2014 Feb 13;10(2):e1003931. doi: 10.1371/journal.ppat.1003931 (PMC3923773; doi:10.1371/journal.ppat.1003931)
Supplement: Figure S3 — The interaction between purified mosGCTL-3 and the E proteins of 4 dengue serotypes by ELISA. (A) The 4 DENV Envelope genes (DENV-1, Hawaii; DENV-2, New Guinea C; DENV-3, Guangdong; DENV-4, H241) with a FLAG tag were cloned into pMT/Bip/V5-His A DNA vector. The recombinant DENV E proteins were expressed in Drosophila S2 cells. The interaction was determined by ELISA. In the study, the purified mosGCTL-3 (2 ug) was coated on the wells. BSA served as a mock control. The S2 expressed DENV E proteins was then respectively incubated in the wells (the amount balanced by Western-blotting shown in Figure S3B). An anti-FLAG mouse mAb was used to probe the interaction. The experiment was reproduced three times with the similar results. (B) Determination of the loading E proteins in ELISA. The DENV E proteins expressed in S2 cells were measured by Western-blotting with anti-FLAG mAb. (PDF) [file ppat.1003931.s003.pdf]

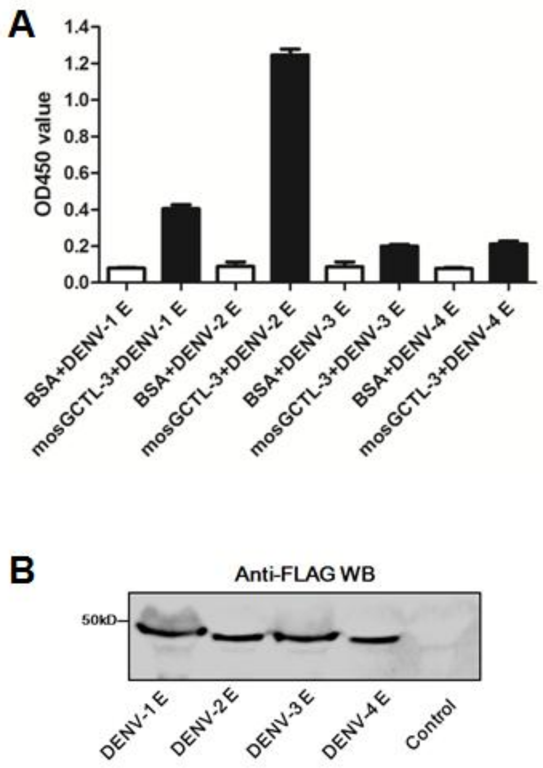

Figure S3. The interaction between purified mosGCTL-3 and the E proteins of 4 dengue serotypes by ELISA.

(A) The 4 DENV Envelope genes (DENV-1, Hawaii; DENV-2, New Guinea C; DENV-3, Guangdong; DENV-4, H241) with a FLAG tag were cloned into pMT/Bip/V5-His A DNA vector. The recombinant DENV E proteins were expressed in *Drosophila* S2 cells. The interaction was determined by ELISA. In the study, the purified mosGCTL-3 (2 ug) was coated on the wells. BSA served as a mock control. The S2 expressed DENV E proteins was then respectively incubated in the wells (the amount balanced by Western-blotting shown in Figure S3B). An anti-FLAG mouse mAb was used to probe the interaction. The experiment was reproduced three times with the similar results.

(B) Determination of the loading E proteins in ELISA. The DENV E proteins expressed in S2 cells were measured by Western-blotting with anti-FLAG mAb.
