# Supplementary material for: Transmission-Blocking Antibodies against Mosquito C-Type Lectins for Dengue Prevention
Source: PLoS Pathog. 2014 Feb 13;10(2):e1003931. doi: 10.1371/journal.ppat.1003931 (PMC3923773; doi:10.1371/journal.ppat.1003931)
Supplement: Figure S4 — The expression of mosGCTL-3 during DENV-2 infection of A. aegypti tissues. (A) DENV-2 distribution in selected A. aegypti tissues. The virus was inoculated into mosquito thorax by microinjection and DENV-2 load was determined by qPCR and normalized by A. aegypti actin. (B–E) The regulation of mosGCTL-3 by DENV-2 infection in mosquito tissues, including whole body (B), salivary glands (C), midgut (D), and hemolymph (E). Total RNA was isolated from various tissues or whole mosquitoes at time courses. Each group included at least 9 individual tissues or mosquitoes. (PDF) [file ppat.1003931.s004.pdf]

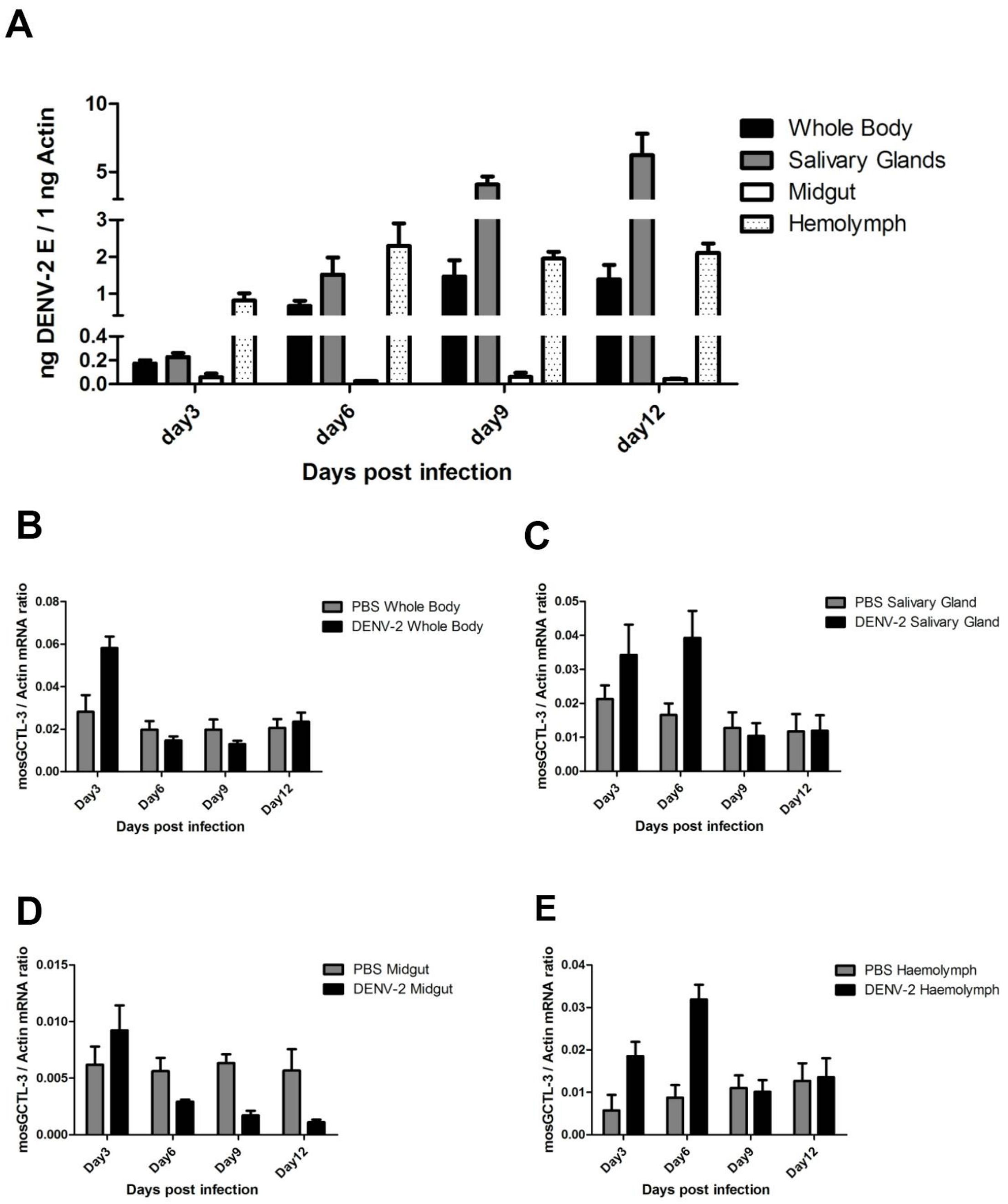

Figure S4. The expression of *mosGCTL-3* during DENV-2 infection of *A. aegypti* tissues.

(**A**) DENV-2 distribution in selected *A. aegypti* tissues. The virus was inoculated into mosquito thorax by microinjection and DENV-2 load was determined by qPCR and normalized by *A. aegypti actin*.

(**B-E**) The regulation of *mosGCTL-3* by DENV-2 infection in mosquito tissues, including whole body (B), salivary glands (C), midgut (D), and hemolymph (E). Total RNA was isolated from various tissues or whole mosquitoes at time courses. Each group included at least 9 individual tissues or mosquitoes.

(**A-E**) The viral load and *mosGCTL-3* expression was detected by RT-QPCR and normalized with *A. aegypti actin* (*AAEL011197*). 1,000 M.I.D.$_{50}$ DENV-2 was inoculated into each mosquito. Data were shown as the mean ± standard error (SEM). The result was combined from 2 independent experiments.
