# Supplementary material for: Transmission-Blocking Antibodies against Mosquito C-Type Lectins for Dengue Prevention
Source: PLoS Pathog. 2014 Feb 13;10(2):e1003931. doi: 10.1371/journal.ppat.1003931 (PMC3923773; doi:10.1371/journal.ppat.1003931)
Supplement: Figure S5 — Validation of mosGCTLs polyclonal antibodies. (A–B) mosGCTLs antisera were produced by immunization of the E.coli-expressed antigens in rabbits. The mosGCTLs polyclonal antibodies were validated by detection of the recombinant mosGCTLs proteins from S2 cells (A) and the native proteins in mosquito lysate (B). (PDF) [file ppat.1003931.s005.pdf]

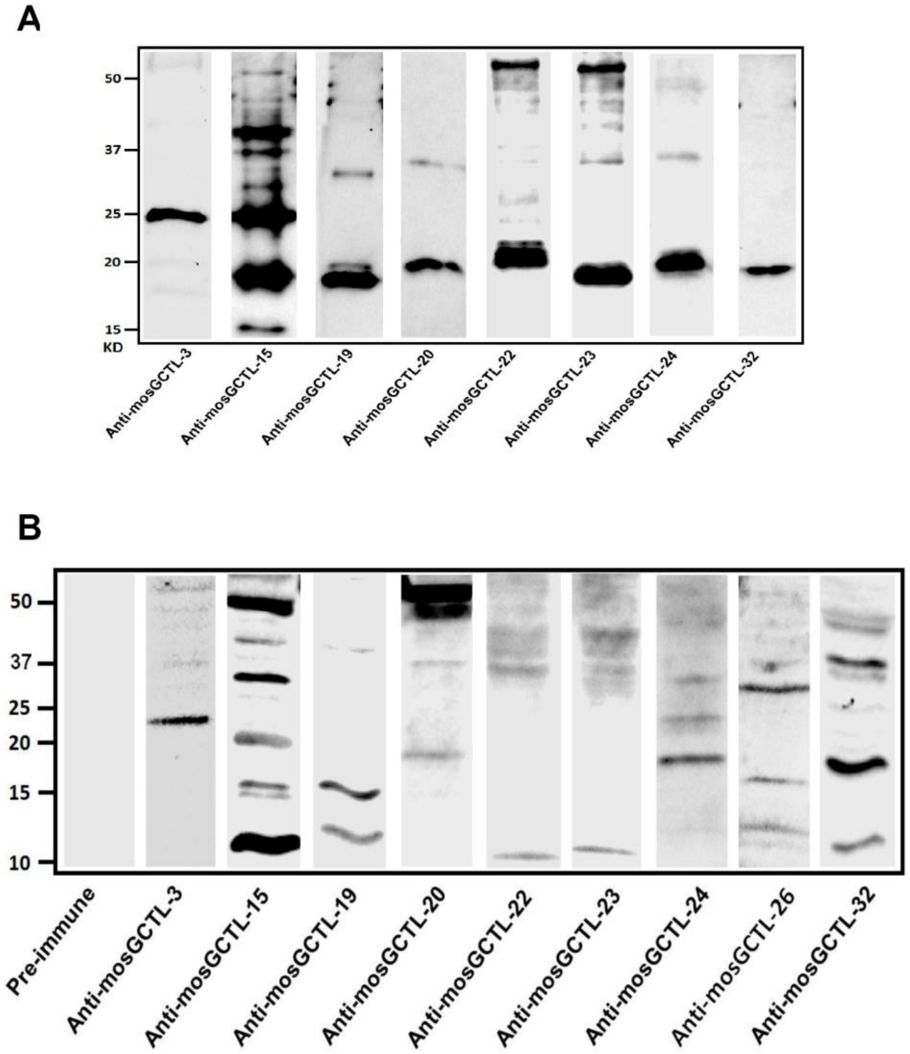

Figure S5. Validation of mosGCTLs polyclonal antibodies.

(**A**-**B**) mosGCTLs antisera were produced by immunization of the *E.coli*-expressed antigens in rabbits. The mosGCTLs polyclonal antibodies were validated by detection of the recombinant mosGCTLs proteins from S2 cells (A) and the native proteins in mosquito lysate (B).
