# Supplementary material for: Transmission-Blocking Antibodies against Mosquito C-Type Lectins for Dengue Prevention
Source: PLoS Pathog. 2014 Feb 13;10(2):e1003931. doi: 10.1371/journal.ppat.1003931 (PMC3923773; doi:10.1371/journal.ppat.1003931)
Supplement: Table S1 — The role of 36 mosGCTL genes in DENV-2 infection in A. aegypti . Double-stranded RNAs (dsRNA) against mosGCTLs were synthesized and microinjected into mosquito thorax to knock down the target gene. After 3 days post dsRNA microinjection, 10 M.I.D50 (Mosquito Infective Dose 50%) DENV-2 was injected into the mosquitoes. After 6 days, mosquitoes were sacrificed and the virus burden assessed. 9 of the 33 genes showed a significant decrease of the virus burden (p<0.05). Statistical analysis was done with the Mann-Whitney test. (PDF) [file ppat.1003931.s006.pdf]

Table S1. The role of 36 *mosGCTL* genes in DENV-2 infection in *A. aegypti*.

Gene Symbol	*Gene ID*	*DENV burden 6dpi (Fold/Mock)*	*p value*
mosGCTL-1	AAEL000563	1.02±1.13	
mosGCTL-2	AAEL000533	0.79±0.31	
mosGCTL-3	AAEL000535	0.26±0.21	$p<0.0001$
mosGCTL-4	AAEL000543	1.09±0.67	
mosGCTL-5	AAEL000556	0.71±0.65	
mosGCTL-6	AAEL000283	N/A*	
mosGCTL-7	AAEL002524	1.33±0.91	
mosGCTL-8	AAEL004679	1.06±0.68	
mosGCTL-9	AAEL005482	0.95±0.70	
mosGCTL-10	AAEL005641	1.40±1.41	
mosGCTL-11	AAEL006456	1.21±0.83	
mosGCTL-12	AAEL008299	1.53±0.83	
mosGCTL-13	AAEL008681	0.80±0.65	
mosGCTL-14	AAEL009209	0.94±0.50	
mosGCTL-15	AAEL010992	0.40±0.44	$p<0.005$
mosGCTL-16	AAEL011070	0.88±0.65	
mosGCTL-17	AAEL011079	N/A	
mosGCTL-18	AAEL011402	0.76±0.59	
mosGCTL-19	AAEL011404	0.44±0.35	$p<0.0005$
	AAEL011407	*Similar to AAEL011404 90.8% identity in Nucleotide*	
mosGCTL-20	AAEL011408	0.54±0.25	$p<0.05$
mosGCTL-21	AAEL011446	0.92±0.97	
mosGCTL-22	AAEL011453	0.52±0.26	$p<0.0005$

mosGCTL-23	AAEL011455	0.51 ±0.33	*p<0.0005*
mosGCTL-24	AAEL011607	0.59 ±0.49	*p<0.0001*
mosGCTL-25	AAEL011609	N/A	
mosGCTL-26	AAEL017265	0.51 ±0.23	*p<0.0005*
mosGCTL-27	AAEL011612	0.71 ±0.65	
mosGCTL-28	AAEL011616	1.41 ±0.87	
mosGCTL-29	AAEL011619	1.48 ±1.09	
mosGCTL-30	AAEL011621	1.66 ±1.25	
mosGCTL-31	AAEL011622	0.73 ±0.34	
mosGCTL-32	AAEL012353	0.40 ±0.51	*p<0.0005*
	AAEL014382	*Similar to AAEL011607 98.3% identity in Nucleotide*	
mosGCTL-33	AAEL014385	1.19 ±0.61	
	AAEL014390	*Similar to AAEL011619 98.9% identity in Nucleotide*	

Double-stranded RNAs (dsRNA) against *mosGCTLs* were synthesized and microinjected into mosquito thorax to knock down the target gene. After 3 days post dsRNA microinjection, 10 $M.I.D_{50}$ (Mosquito Infective Dose 50%) DENV-2 was injected into the mosquitoes. After 6 days, mosquitoes were sacrificed and the virus burden assessed. 9 of the 33 genes showed a significant decrease of the virus burden ($p<0.05$). Statistical analysis was done with the *Mann-Whitney* test.

* N/A, gene not analyzed.
