# Supplementary material for: Transmission-Blocking Antibodies against Mosquito C-Type Lectins for Dengue Prevention
Source: PLoS Pathog. 2014 Feb 13;10(2):e1003931. doi: 10.1371/journal.ppat.1003931 (PMC3923773; doi:10.1371/journal.ppat.1003931)
Supplement: Table S2 — The specificity of the dsRNA-mediated silencing among the 9 mosGCTL genes. The number represents the ratio of the mosGCTL mRNA amount between mosGCTL-dsRNA and GFP-dsRNA treated mosquitoes. The mosGCTLs dsRNA were inoculated into mosquitoes respectively. GFP dsRNA served as mock control. The mosquitoes were sacrificed at 9 days after dsRNA inoculation. The amount of mosGCTL mRNA was determined by qPCR and normalized by A. aegypti actin. (PDF) [file ppat.1003931.s007.pdf]

Table S2. The specificity of the dsRNA-mediated silencing among 9 *mosGCTL* genes.

Detection of *mosGCTLs* mRNA

dsRNA mediated silencing	mosGCTL-3	mosGCTL-15	mosGCTL-19	mosGCT-20	mosGCTL-22	mosGCTL-23	mosGCTL-24	mosGCTL-26	mosGCTL-32
**mosGCTL-3 RNAi**	0.28*,**	0.61	0.80	0.75	1.00	0.68	0.93	0.63	0.77
**mosGCTL-15RNAi**	1.33	0.28	1.10	1.01	1.03	0.73	0.70	0.67	0.98
**mosGCTL-19RNAi**	0.99	0.89	0.25	1.57	0.69	0.63	0.65	0.61	0.41***
**mosGCTL-20RNAi**	1.11	1.20	0.56	0.21	0.71	0.83	0.92	0.88	1.20
**mosGCTL-22RNAi**	0.72	0.55	0..69	1.07	0.22	0.42	0.43	0.27	0.45
**mosGCTL-23RNAi**	1.04	1.00	1.49	0.28	0.34	0.15	0.30	0.33	0.49
**mosGCTL-24RNAi**	1.08	1.12	0.98	0.79	0.63	0.69	0.21	1.05	1.03
**mosGCTL-26RNAi**	0.89	0.74	0.56	0.71	0.58	0.68	0.72	0.08	0.81
**mosGCTL-32RNAi**	1.09	1.33	1.71	0.97	0.75	0.77	0.71	0.95	0.23

* The number represents the ratio of the *mosGCTL* mRNA amount between *mosGCTL*-dsRNA and *GFP*-dsRNA treated mosquitoes. The *mosGCTLs* dsRNA were inoculated into mosquitoes respectively. *GFP* dsRNA served as mock control. The mosquitoes were sacrificed at 9 days after dsRNA inoculation. The amount of *mosGCTL* mRNA was determined by qPCR and normalized by *A. aegypti actin*.

** The column in deep gray: Silencing effect by its own dsRNA inoculation.

*** The column in light gray: Cross-silencing effect with ratio<0.5 (more than 2-fold decrease).
