# Supplementary material for: Transmission-Blocking Antibodies against Mosquito C-Type Lectins for Dengue Prevention
Source: PLoS Pathog. 2014 Feb 13;10(2):e1003931. doi: 10.1371/journal.ppat.1003931 (PMC3923773; doi:10.1371/journal.ppat.1003931)
Supplement: Table S3 — Primers and probes for qPCR, dsRNA synthesis and gene cloning. (PDF) [file ppat.1003931.s008.pdf]

Table S3. Primers and probes for qPCR, dsRNA synthesis and gene cloning

**The primers for SYBR Green RT-QPCR**	**Upper primer**	**Lower primer**	
SYBR mosGCTL-1	TGTATGGCGTTGCACTACT	CTTGAAGTTCTCTATGCAGG	
SYBR mosGCTL-2	ATGGCTCTTTCATTATATCT	CACAACATTTGGGAAGCAGAA	
SYBR mosGCTL-3	GATTTTTGCTTTGCTATATGTTGCTT	GGTCCTCTTGTTTGGGATGAAG	
SYBR mosGCTL-4	CTGGTCAGGTATGCCAAT	CACTCGTTATCATTCCAAG	
SYBR mosGCTL-5	ATGCCTGGAAAACCGGAC	CTTCCTCTCGCACACAAA	
SYBR mosGCTL-6	GTTCTCGTACATTCATCTCATG	CTTGAACCAGTTAGCTCGAA	
SYBR mosGCTL-7	GAGTAGCAGACTGATGACGTTT	CTTTGCCGCTCTTCACAAA	
SYBR mosGCTL-8	GTTCGTCAAGAAAAACTGG	GTAGATAGAGGATCCAACAA	
SYBR mosGCTL-9	GGTACCCCATTCGGACACTT	TGGTTTTAACGATCTGCTTTTG	
SYBR mosGCTL-10	CGTGGAGATACGACACAT	ACCTCCTTCCCTACATCT	
SYBR mosGCTL-11	ACTGCGTTCACATTCAGT	TCACCGACAATATTTTTCGC	
SYBR mosGCTL-12	CGACAACCGGAAGGACGA	AAATCGGCCTCTCGCAAA	
SYBR mosGCTL-13	ATGCAGTTCAAAATCGTTC	TGTACTTGTTCAGCGGAAG	
SYBR mosGCTL-14	CCGAAGTCAGCCGGACAAT	TCTCACAAACCGGCACCAA	
SYBR mosGCTL-15	GTGTCAAGGAAGAGAATCGCG	ACTTCATCTCGAGTTCTGCCG	
SYBR mosGCTL-16	CCTAACGGCTGGGGATACA	GATTCGGTCGTCTGGTTGATG	
SYBR mosGCTL-17	GATGCAGTTTACCAACTG	CTAAGGCAGTGGTTCTACA	
SYBR mosGCTL-18	CAATCGGGCGGTGTTTAGTTTT	CTCGCGAAGGCCTCAGTAGC	
SYBR mosGCTL-19	GTTATTTTATCAGTTATGTTGC	GACGCCTCAAACCAATTGG	
SYBR mosGCTL-20	CGTCTTGTTCATCGACTAAACC	CGACGATTCGAACCAATTGG	
SYBR mosGCTL-21	ACACCGATCCATTCAATCG	GCTTAATCCCTTTCTCAC	
SYBR mosGCTL-22	CTCACAATTGTGTCGAAATG	CCGAAATTCACTAATGCAG	
SYBR mosGCTL-23	TGCGTGGAGCTGACCTATC	CTACAGACATTGTCGTTCG	
SYBR mosGCTL-24	AACGCTGCAGTTATGCGT	GTTCAACCTTTCACAAACG	
SYBR mosGCTL-25	AAATATGTGCATGGTGGCTGTG	CTATTCCCCGGTGGTTCC	
SYBR mosGCTL-26	CATGACAGGATACTTCAAC	GACCAGTTTGGAGTGACAG	
SYBR mosGCTL-27	GAAGAAGGCAGCTTCTTCTG	TTCCGTCTCGATTCCACAG	
SYBR mosGCTL-28	TGCACCGCTCTTATCGGACTTC	CGTATGCCACGGGTAATCTCCA	
SYBR mosGCTL-29	CAATGCTGGCGCAAATGAAC	GATTGGACTGACAGATTCAC	
SYBR mosGCTL-30	CTGCCCGGGAAGAACAATCAAA	ACTCGGGGCGGCACATCC	
SYBR mosGCTL-31	ATGGCGACAGTGGTGGTGGATT	TTGCCGCTGTAAACGATGTGAT	
SYBR mosGCTL-32	TCGAGGCATCCGAATACTGC	CGGCCTTGGCCTCCTTTACT	
SYBR mosGCTL-33	GAGAAAATGAACACTGTCTC	CGACCAGTGTGGACTGACAGT	
**The primers for Taqman RT-QPCR**	**Upper primer**	**Lower primer**	**Probe (for Taqman QPCR)**
DENV-1 Envelop gene	GACACCACACCCTTTGGACAA	CACCTGGCTGTCACCTCCAT	FAM-AGAGGGTGTTTAAAGAGAAAGTTGACACGCG-TAMRA
DENV-2 Envelop gene	CATTCCAAGTGAGAATCTCTTTGTCA	CAGATCTCTGATGAATAACCAACG	FAM-ATGCTGAAACGCGAGAGAAACCGC-TAMRA
DENV-3 Envelop gene	GGGAAAACCGTCTATCAATA	CGCCATAACCAATTTCATTGG	FAM-CACAGTTGGCGAAGAGATTCTCAAGAGGA-TAMRA
DENV-4 Envelop gene	TGAAGAGATTCTCAACCGGAC	AATCCCTGCTGTTGGTGGG	FAM-TCATCACGTTTTTGCGAGTCCTTTCCA-TAMRA
Aedes Actin	GAACACCCAGTCCTGCTGACA	TGCGTCATCTTCTCACGGTTAG	FAM-AGGCCCCGCTCAACCCGAAG-TAMRA
**The primers for double-strand RNA synthesis**	**Upper primer**	**Lower primer**	
dsRNA GFP	TAATACGACTCACTATAGGGGTGAGCAAGGGCGAGGAG	TAATACGACTCACTATAGGGCATGATATAGACGTTGTGGCTGTT	
dsRNA mosGCTL-1	TAATACGACTCACTATAGGGTTTGGCCGTTCGAAAATCTGAA	TAATACGACTCACTATAGGGGGTTCTACGCGCTCGCAAATC	
dsRNA mosGCTL-2	TAATACGACTCACTATAGGGGACATGCGACAACGACAACAGATT	TAATACGACTCACTATAGGGAGCGCACACCACGTCATTCCAGT	
dsRNA mosGCTL-3	TAATACGACTCACTATAGGGATGGATATGCGACTGGCAGT	TAATACGACTCACTATAGGGGCCACAACGACTCAGAAA	
dsRNA mosGCTL-4	TAATACGACTCACTATAGGGATGCACACATATAGCCGATG	TAATACGACTCACTATAGGGGTACCTTCCTCTGCCAGATC	
dsRNA mosGCTL-5	TAATACGACTCACTATAGGGGCTACTTCTGTTCACTTTGG	TAATACGACTCACTATAGGGGTGCTCCTTTCCGTCCTTA	
dsRNA mosGCTL-6	TAATACGACTCACTATAGGGTCCCAGCATTCGAGCTAACTGG	TAATACGACTCACTATAGGGCCTGCGCAGGTGTTATCATTCC	
dsRNA mosGCTL-7	TAATACGACTCACTATAGGGGTCCACGTTCTTGTCGTT	TAATACGACTCACTATAGGGCACACAGTTCTCGGCTTCTG	
dsRNA mosGCTL-8	TAATACGACTCACTATAGGGCGAATACTGCAAATGCTAC	TAATACGACTCACTATAGGGGCTTTTTCACACGCTACATTGG	
dsRNA mosGCTL-9	TAATACGACTCACTATAGGGCGGCGCCTATGCGTACTGTAAAAC	TAATACGACTCACTATAGGGAATGCAATCCGTATCACTTGTGCT	
dsRNA mosGCTL-10	TAATACGACTCACTATAGGGTGTATTGCTCAGCAGGTG	TAATACGACTCACTATAGGGCGTATCTCCACGCAGTGTTCAA	
dsRNA mosGCTL-11	TAATACGACTCACTATAGGGCAATTGATCAGACCAACTG	TAATACGACTCACTATAGGGGATCGTCATTCCACTGGAA	
dsRNA mosGCTL-12	TAATACGACTCACTATAGGGCAAAGCGACCGAGTACTG	TAATACGACTCACTATAGGGCACCACGATCCACTTGAA	
dsRNA mosGCTL-13	TAATACGACTCACTATAGGGCTGTTGGCGGCTGCGTTGGT	TAATACGACTCACTATAGGGAAATGTACTTGCGGGGCTCGTC	
dsRNA mosGCTL-14	TAATACGACTCACTATAGGGATGATGCGCTTCGTTGTT	TAATACGACTCACTATAGGGCCACATGTGAATGCAATGC	
dsRNA mosGCTL-15	TAATACGACTCACTATAGGGGGCGTCCAGTATTTCGTGAG	TAATACGACTCACTATAGGGGTGCGCTGAGGGGAGGTGTTG	
dsRNA mosGCTL-16	TAATACGACTCACTATAGGGATCGAATCAGCCCCAGCAGAAGC	TAATACGACTCACTATAGGGGGTTGTATCCCCAGCCGTTAGG	
dsRNA mosGCTL-17	TAATACGACTCACTATAGGGATGGCTCTTTTACGGTTC	TAATACGACTCACTATAGGGCCGGTTGGTACCACATGTGAA	
dsRNA mosGCTL-18	TAATACGACTCACTATAGGGGATGGTTTGAGCGGGCAGGACT	TAATACGACTCACTATAGGGGAAGGCGGAGCGGGAAGGTTT	
dsRNA mosGCTL-19	TAATACGACTCACTATAGGGGAGGCGTCCGAGTACTGTCACC	TAATACGACTCACTATAGGGCTCAATTCGCAGATGGCGTAGT	
dsRNA mosGCTL-20	TAATACGACTCACTATAGGGCCACATAGCAACTTATGC	TAATACGACTCACTATAGGGCTCCTCTATGCACGTCGT	
dsRNA mosGCTL-21	TAATACGACTCACTATAGGGCTTCTTTCGCTAACAACAG	TAATACGACTCACTATAGGGCCAATGTGCAATCTCCAGA	
dsRNA mosGCTL-22	TAATACGACTCACTATAGGGATGCATAAAGCACTGCTG	TAATACGACTCACTATAGGGCTGTTTTGCCAATTCCAG	
dsRNA mosGCTL-23	TAATACGACTCACTATAGGGCAACATGAGAGTGCTCGT	TAATACGACTCACTATAGGGCACTCCATGTCGTTCCAAAA	
dsRNA mosGCTL-24	TAATACGACTCACTATAGGGTCGGCACTCTCAAACTCACTG	TAATACGACTCACTATAGGGTAACTGCAGCGTTCGGTTC	
dsRNA mosGCTL-25	TAATACGACTCACTATAGGGAACGCAACAGCTAGCTTCGGATGA	TAATACGACTCACTATAGGGGTCGGAAGGTTGTTCGGTTTGTCA	
dsRNA mosGCTL-26	TAATACGACTCACTATAGGGGTCTGTTGCATGCTTGCTAC	TAATACGACTCACTATAGGGGTTGAAGTATCCTGTCATG	
dsRNA mosGCTL-27	TAATACGACTCACTATAGGGGCAGTTGTGCGGATTGTT	TAATACGACTCACTATAGGGCCGTTAGAGATCCAGAAGAA	
dsRNA mosGCTL-28	TAATACGACTCACTATAGGGGGCAAACGCGCCTATTGTATCCAG	TAATACGACTCACTATAGGGGCGTTGACCAATGGCTTGACACAG	
dsRNA mosGCTL-29	TAATACGACTCACTATAGGGCCCAATTGTTGACGAGGATG	TAATACGACTCACTATAGGGCCGATCTCAAGACAGTGTTC	
dsRNA mosGCTL-30	TAATACGACTCACTATAGGGATGTGCCGCCCCGAGTTGAC	TAATACGACTCACTATAGGGTGCTTATTTCCTGTGACCGCTACG	
dsRNA mosGCTL-31	TAATACGACTCACTATAGGGATTGGCTTTTTGCACCGCTCTTAT	TAATACGACTCACTATAGGGTCCGCTGTGAACGATTATTGAAC	
dsRNA mosGCTL-32	TAATACGACTCACTATAGGGTGCGGTTGGCCATAGTTGAT	TAATACGACTCACTATAGGGAGCCGCCTTTTCGTAGTTTTC	
dsRNA mosGCTL-33	TAATACGACTCACTATAGGGATGGAAGCATGGAGACTGTGTC	TAATACGACTCACTATAGGGGTACATCATTCCATTTTACAC	
**Primers for 5' RACE**	**Outer primer**	**Inner primer**	
mosGCTL-3 specific 5' RACE primers	GCCACAACGACTCAGAAA	GTAGGCGATTTCCACGCAATC	
**Primers for cloning into pET28a(+)**	**Upper primer**	**Lower primer**	
mosGCTL-3 (His tag in both N and C-terminal)	TCTCTAGCTAGCCAGCCCATATGCAGTGAT	TATCCGCTCGAGAAACTCCTGGATTGAAT	
mosGCTL-15 (His tag in both N and C-terminal)	CTGCCGGCTAGCCAACGTATCACCACAATCC	TATACCCTCGAGCCCCTGCGCCGACGA	
mosGCTL-19 (His tag in both N and C-terminal)	CGCCTAGCTAGCCAATTCAACTTCCAT	TCACCGCTCGAGGATGCAATACCTCAAT	
mosGCTL-20 (His tag in both N and C-terminal)	TCATCGGCTAGCTCATCGACTAAACCAAAAT	TTATCCCTCGAGAAACTCCTCTATGCACGTC	
mosGCTL-22 (His tag in both N and C-terminal)	CGCCTAGCTAGCATAAGCGAATACTTTATTCCT	TCACCGCTCGAGCCGAAATTCACTAATGCA	
mosGCTL-23 (His tag in both N and C-terminal)	CGCCTAGCTAGCGATAGACGATTCTTCATACCCA	TTATCCCTCGAGCAGACATTGTCGTTCGA	
mosGCTL-24 (His tag in both N and C-terminal)	TCATCGGCTAGCGATCCCCTAAGAAATCAGAC	TTATCCCTCGAGGTTCAACCTTTCACAAA	
mosGCTL-26 (His tag in both N and C-terminal)	TCTCTAGCTAGCACACATTTTTGCTCCC	TATTCGAAGCTTCGACCAGTTTGGAGTGAC	
mosGCTL-32 (His tag in both N and C-terminal)	CGCCTAGCTAGCCAATCCAAGTATCAAAT	TATCCGCTCGAGAAACTCTTGGATGC	
**Primers for cloning into pMT/BiP/V5-His/A**	**Upper primer**	**Lower primer**	
mosGCTL-3 (V5 tag in C-terminal)	TATAGGGGTACCTCAGCCCATATGCAGTGAT	CGCTGCTCTAGAAAACTCCTGGATTGAATC	
mosGCTL-15 (V5 tag in C-terminal)	TATTGGGGTACCCCATGAATCCCTACTCGCC	TATTGCTCTAGACCCCTGCGCCGACG	
mosGCTL-19 (V5 tag in C-terminal)	TCCTGAAGATCTCAATTCAACTTCCATATCGCTT	TCCTGATCTAGAGATGCAATACCTCAATTCGCA	
mosGCTL-20 (V5tag in C-terminal)	TACTGGGGTACCTTCATCGACTAAACCAAAAT	CACTGCTCTAGAAAACTCCTCTATGCACGTC	
mosGCTL-22 (V5 tag in C-terminal)	TCCTGAAGATCTATAAGCGAATACTTTATTCCT	TCCTGATCTAGACCGAAATTCACTAATGCAGT	
mosGCTL-23 (V5tag in C-terminal)	TCCTGAAGATCTGATAGACGATTCTTCATACCCA	TCCTGATCTAGACAGACATTGTCGTTCGATTTC	
mosGCTL-24 (V5 tag in C-terminal)	TCCTGAAGATCTGATCCCCTAAGAAATCAGAC	TCCTGATCTAGAGTTCAACCTTTCACAAACGAA	
mosGCTL-26 (V5 tag in C-terminal)	TCCTGAAGATCTACACATTTTTGCTCCCCAATT	TCCTGATCTAGACGACCAGTTTGGAGTGACAGA	
mosGCTL-32 (V5 tag in C-terminal)	TCATGGGGTACCGCAATCCAAGTATCAAAT	CGCTGCTCTAGAAAACTCTTGGATGC	
DENV-2 Envelop gene (3×FLAG tag in N-terminal)	CGAAGATCTGCCACCATGGACTACAAAGACCATGACGGTGATTATAAAGATCATGACATCGATTACAAGGATGACGATGACAAGATGCGTTGCATAGGAATA	AGACTCGAGTCAATGGTGATGGTGATGATGTTGGCCGATAGAACTTC	
